# Patient‐Derived Organoids from Colorectal Cancer with Paired Liver Metastasis Reveal Tumor Heterogeneity and Predict Response to Chemotherapy

**DOI:** 10.1002/advs.202204097

**Published:** 2022-09-04

**Authors:** Shaobo Mo, Peiyuan Tang, Wenqin Luo, Long Zhang, Yaqi Li, Xiang Hu, Xiaoji Ma, Yikuan Chen, Yichao Bao, Xingfeng He, Guoxiang Fu, Xiaoya Xu, Xinxin Rao, Xiaomeng Li, Ruoyu Guan, Shengzhi Chen, Yun Deng, Tao Lv, Peiyuan Mu, Qiang Zheng, Simin Wang, Fangqi Liu, Yiwei Li, Weiqi Sheng, Dan Huang, Chen Hu, Jianjun Gao, Zhen Zhang, Sanjun Cai, Hans Clevers, Junjie Peng, Guoqiang Hua

**Affiliations:** ^1^ Department of Colorectal Surgery Fudan University Shanghai Cancer Center Fudan University Shanghai 200032 P. R. China; ^2^ Department of Oncology Shanghai Medical College Fudan University Shanghai 200032 P. R. China; ^3^ Institute of Radiation Medicine Shanghai Medical College Fudan University Shanghai 200032 P. R. China; ^4^ Cancer Institute Fudan University Shanghai Cancer Center Shanghai 200032 P. R. China; ^5^ Research and Early Development D1Med Technology (Shanghai) Inc Shanghai 200235 P. R. China; ^6^ Department of Radiation Oncology Fudan University Shanghai Cancer Center Fudan University Shanghai 200032 P. R. China; ^7^ Department of Pathology Fudan University Shanghai Cancer Center Shanghai 200032 P. R. China; ^8^ Department of Radiology Fudan University Shanghai Cancer Center Shanghai 200032 P. R. China; ^9^ Sidney Kimmel Comprehensive Cancer Center Johns Hopkins University School of Medicine Baltimore MD 21218 USA; ^10^ Hubrecht Institute KNAW and University Medical Center Utrecht P.O. Box 85500 Utrecht 3584 CT The Netherlands

**Keywords:** chemotherapy response, colorectal cancer liver metastasis, patient‐derived organoid, prognosis prediction, tumor heterogeneity

## Abstract

There is no effective method to predict chemotherapy response and postoperative prognosis of colorectal cancer liver metastasis (CRLM) patients. Patient‐derived organoid (PDO) has become an important preclinical model. Herein, a living biobank with 50 CRLM organoids derived from primary tumors and paired liver metastatic lesions is successfully constructed. CRLM PDOs from the multiomics levels (histopathology, genome, transcriptome and single‐cell sequencing) are comprehensively analyzed and confirmed that this organoid platform for CRLM could capture intra‐ and interpatient heterogeneity. The chemosensitivity data in vitro reveal the potential value of clinical application for PDOs to predict chemotherapy response (FOLFOX or FOLFIRI) and clinical prognosis of CRLM patients. Taken together, CRLM PDOs can be utilized to deliver a potential application for personalized medicine.

## Graphical abstract

A living biobank of patient‐derived organoid (PDO) is derived from primary colorectal cancer and paired liver metastatic lesions, which captures intra‐ and interpatient molecular fingerprint heterogeneity. PDO chemosensitivity measured in vitro may be used as a predictive tool for clinical chemotherapeutic efficacy, and guide the formulation of precise treatment strategies for colorectal cancer patients with liver metastases.

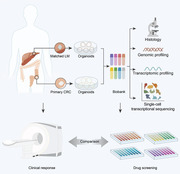

## 1 Introduction

Colorectal cancer (CRC) has become the third most common malignant tumor worldwide.^[^ ^1^ ^]^ About 20% of newly diagnosed CRC patients will have synchronous liver metastases (LM) and at least half of patients who develop postoperative metastatic disease will have liver metastases.^[^ ^2^ ^]^ Surgical resection and systemic chemotherapy are considered the mainstay of therapy for colorectal cancer liver metastasis (CRLM).^[^ ^3^, ^4^ ^]^ However, the standardized treatment mode is still under constant exploration and improvement considering the complexity of CRLM, and the effectiveness of adjuvant therapy remains controversial.^[^ ^5^ ^]^ In addition, clonal heterogeneity is a characteristic of most human cancers,^[^ ^6^ ^]^ which may complicate the choice of optimal adjuvant therapy for CRLM patients.

5‐fluorouracil (5‐FU), oxaliplatin, and irinotecan (CPT11) are main clinical first‐line chemotherapy drugs.^[^ ^7^ ^]^ It has been shown that the median overall survival (OS) and progression‐free survival (PFS) of CRLM patients treated with 5‐FU/leucovorin (5‐FU/LV) plus oxaliplatin (FOLFOX) or irinotecan (FOLFIRI) are higher than those received 5‐FU/LV alone.^[^ ^8^ ^]^ Even so, a considerable proportion of CRLM patients do not benefit from chemotherapy but bear the resulting side effects.^[^ ^9^ ^]^ A few clinical indicators may help to provide prognostic information, but most of the recommended biomarkers have not been used to predict chemotherapy response.^[^ ^10^, ^11^ ^]^ Preclinical models, such as cell lines or patient‐derived tumor xenografts (PDTXs), have limited predictive response to treatment due to long duration of experiment, poor scalability, and low success rate.^[^ ^12^ ^]^ Briefly, there is a lack of personalized cancer treatment for chemotherapy and new prediction model is urgently needed.

**Scheme 1:**
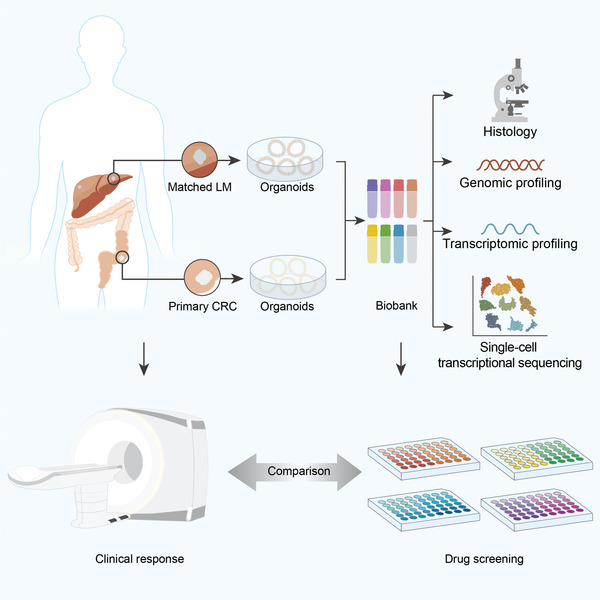
Graphical summary of the study concept. A living biobank with 50 CRLM organoids derived from primary tumors and paired liver metastatic lesions was constructed and comprehensively analyzed from the multi omics levels (histopathology, genome, transcriptome, and single‐cell sequencing). Moreover, PDOs manifest potential to predict chemotherapy response and clinical prognosis of CRLM patients.

Patient‐derived organoid (PDO) can be obtained from individual patients with high success rate, short culture period and unlimited expansion, which can highly recapitulate physiology of the original tumor.^[^ ^13^ ^]^ Accumulating studies have shown that PDO can not only be used to explore tumor biological characteristics in basic research, but also be used as a preclinical model to predict patients’ response to treatment.^[^ ^13^, ^14^, ^15^, ^16^, ^17^ ^]^ Nevertheless, organoids derived from primary cancers and paired metastatic lesions remain limited, given its rarity and difficulty of obtaining biological specimens. Our CRLM organoid biobank complements other published cohorts by comprehensively representing characteristics of the biobank from multiple perspectives of histopathology, genome, transcriptome and single cell sequencing. We also evaluated whether CRLM PDOs can effectively predict chemotherapy response and clinical prognosis (**Scheme** **1**).

## 2 Results

### 2.1 Establishment of a Living CRLM Organoid Biobank

**Figure 1:**
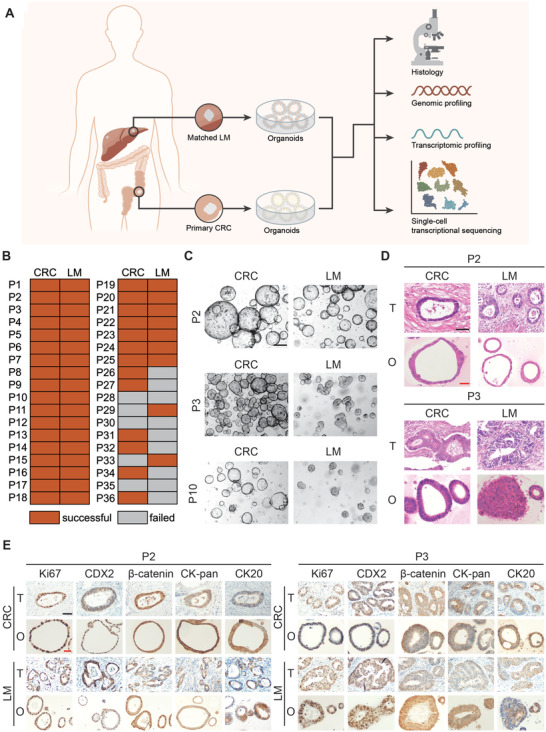
Study design and histopathological characterization of PDOs from CRLM patients. A) Establishment of CRLM patient derived organoid library and multiomics analysis of CRLM organoids (histopathology, genomic profiling, transcriptomic profiling and single‐cell transcriptional sequencing). B) PDOs growth success rate derived from CRC and LM tissue in different CRLM patients. C) The morphology of CRLM organoids with three typical characteristics in bright field. Both CRC and LM organoids from P2 CRLM patient showed thin‐walled cystic structures (top); The CRC organoids from P3 CRLM patient showed thick‐walled cystic structures, while LM organoids showed irregular solid/compact structures (middle); The CRC organoids from P10 CRLM patient presented thin‐walled cystic structures, while LM organoids presented solid spherical structures (bottom). Black scale bar, 200 µm. D) H&E staining comparing CRLM organoids with corresponding primary tumors. (T, primary tumors; O, CRLM organoids). Black scale bar, 200 µm. Red scale bar, 100 µm. E) Immunohistochemistry staining of ki‐67, CDX2, *β*‐catenin, CK‐pan, and CK20 on CRLM organoids and corresponding primary tumors. (T, primary tumors; O, CRLM organoids). Black scale bar, 200 µm. Red scale bar, 100 µm. See also Table S1 and Figures S1–S4 (Supporting Information).

In this study, we successfully constructed a CRLM PDO biobank in vitro by obtaining surgical tissue samples of primary CRC and matched LM tissues from synchronous CRLM patients who underwent simultaneous enterohepatectomy in our center (**Figure** **1**A). From September 2018 to June 2020, a total of 36 CRLM patients were enrolled. 72 surgical tissue samples (36 primary CRC tissues and 36 matched LM tissues) were obtained, and 58 organoids were successfully cultured (80.6% overall success rate), including 31 CRC organoids (86.1% success rate) and 27 LM organoids (75.0% success rate), which was in line with previous reports.^[^ ^13^, ^16^ ^]^ Finally, 50 CRLM organoids from 25 patients were included for subsequent research and analysis, excluding no matched cultures (Figure 1B and Figure S1A, Supporting Information).

CRLM organoids were cultured with organoid culture medium as previously described.^[^ ^18^, ^19^ ^]^ Demographic and clinicopathological characteristics of 25 CRLM patients were presented in Table S1 (Supporting Information). The pathological type of most tumors was adenocarcinoma, and one case was mucinous adenocarcinoma. Most tumors were moderately differentiated, and 4 cases were poorly differentiated. We found that clinical parameters did not affect PDO culture success, interestingly, there were some differences in the causes of failure between CRC and LM organoids. Further analysis showed that the main reasons for failure in CRC organoids culture were bacterial contamination (5.6%) and failure of passaging or expanding (5.6%), while the mainly failure reasons for LM organoids culture were failure of passaging or expanding (14%), but bacterial contamination only accounting for 2.7% (Figure S1B, Supporting Information).

### 2.2 CRLM Organoids Preserve the Histopathological Structures of Parental Tumors

CRLM organoids preserve the same histopathological features of corresponding tumors, as with gastrointestinal cancer organoids derived from other tissues.^[^ ^13^, ^16^, ^20^, ^21^ ^]^ CRLM organoids show great diversity in growth rate and morphology.^[^ ^22^ ^]^ CRC and LM organoids have similar characteristics, but also retain their own heterogeneity. The morphology of CRLM organoids with three typical characteristics was shown in Figure 1C. Both CRC and LM organoids from P2 CRLM patient showed thin‐walled cystic structures; The CRC organoids from P3 CRLM patient showed thick‐walled cystic structures, while LM organoids showed irregular solid/compact structures; The CRC organoids from P10 CRLM patient presented thin‐walled cystic structures, while LM organoids presented solid spherical structures. Then, H&E staining showed that CRLM PDOs presented patient‐specific heterogeneous morphology, from thin‐walled cystic structures to solid/compact structures, consistent with previous study.^[^ ^14^ ^]^ Meanwhile, CRLM PDOs also retained more subtle cytological features, including large and deep stained nuclei, irregular arrangement, and decreased cytoplasmic ratio (Figure 1D and Figure S2, Supporting Information).

Next, we detected the protein expression of important molecular markers and found that the expression pattern of Ki67, CDX2, *β*‐catenin, CK‐pan, and CK20 in CRLM organoids and parental tumors were consistent (Figure 1E and Figure S3, Supporting Information), as reported previously.^[^ ^14^, ^15^ ^]^ Similarly, the expression levels of MMR related proteins (MLH1, MSH6, MSH2, and PMS2) could also be preserved in PDOs (Figure S4, Supporting Information).

In general, CRLM PDOs preserved the histopathological structures of parental tumors, so as to retain homology with the source individuals and heterogeneity among different individuals.

### 2.3 Genomic Characterization, Tumor Evolution, and Heterogeneity of CRLM Organoids

**Figure 2:**
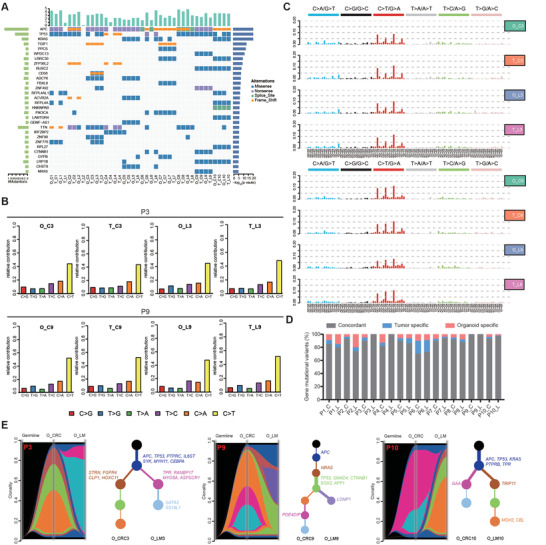
Genomic profiling in CRLM organoids and corresponding primary tumors. A) Overview of somatic mutations found in CRLM organoids and corresponding primary tumors. B,C) Different contributions of point mutation types and mutation characteristics in organoids and corresponding primary tumors from two CRLM patients (P3 and P9 patients) were displayed in bar graphs. D) Bar plots indicate the concordance (%) between the gene mutational variants identified in CRLM organoids and corresponding primary tumors. E) Riverplots generated by SuperFreq analysis showed the clonal evolution of CRLM organoids derived from CRC and paired LM tumor tissues. The *y*‐axis represents the proportion of tumor cells in each subclone. The black area represents germline mutation. The blue region represents somatic mutations detected in all cells of CRC and LM organoids. The remaining color regions represent different subclones present in CRC and/or LM organoids. Next, the situation in the riverplots was visualized as an evolutionary tree (right). Each node represents a subclone (corresponding to different color regions). The thickness of the branch corresponds to the number of mutations obtained in the population. The representative cancer driving genes of each subclone have been displayed and marked corresponding to different color regions. T, tissue; O, organoid; C, CRC; L, LM. See also Figures S5 and S6 (Supporting Information).

Previous studies have reported that PDOs could recapitulate genomic map of the corresponding patient tumors.^[^ ^13^, ^14^, ^15^ ^]^ We performed whole exon sequencing from 10 matched CRLM PDOs and their original tumors. It is found that CRLM PDOs retained the mutation spectrum of the matching primary tumor (**Figure** **2**A). WNT signaling pathway related genes were mutated in all (20/20) CRLM PDOs, including APC, FBXW7, ARID1A, LRP5, CTNNB1, and SOX9.^[^ ^23^ ^]^ Different mutation types of APC gene occurred in 20 (100%) CRLM PDOs, indicating that the expected activation mutation rate of WNT signaling pathway was observed in CRLM PDOs. In addition, the mutation rate of RAS genes (KRAS and NRAS) was 40% (8/20), which was basically consistent with the tissue mutation rate of CRLM patients.^[^ ^23^, ^24^ ^]^ Similarly, using the cancer driver gene database,^[^ ^25^ ^]^ we also found that the mutation pattern of cancer driver genes in CRLM PDOs was highly consistent with paired tumor (Figure S5, Supporting Information), although it was noted that CRLM PDOs could acquire new mutational fingerprints or lose genetic information from parental sources. Specifically, a considerable number of CRLM PDOs reached 100% consistency with their matching tumor, including P9 CRLM patients. Two representative comparisons (P3 and P9 CRLM patients) between examples of CRLM PDOs and the paired primary tumor is shown in Figure 2B,C, revealing that the point mutation types (Figure 2B) and mutation characteristics (Figure 2C) were similar between CRLM PDOs and corresponding tumors. It's observed that CRLM PDOs retained an 88.9% (88.5% for CRC organoids and 89.2% for LM organoids) overlap of the most frequently CRC gene mutational variants^[^ ^23^ ^]^ from the corresponding tumor (Figure 2D).

Then, we used the reported algorithm of tracking clonal evolution^[^ ^26^ ^]^ to investigate whether CRLM PDOs can capture tumor heterogeneity and tumor evolution. It is found that CRC organoids and LM organoids have common early driving mutations, including but not limited to TP53 and APC genes. With clonal evolution, CRC organoids and LM organoids accumulate unique driving mutation patterns, such as FGFR4 (O_CRC3,^[^ ^27^, ^28^ ^]^) and CBL (O_LM10,^[^ ^29^ ^]^) genes, reflecting intra‐patient heterogeneity of primary and metastatic lesions (Figure 2E). Moreover, the pattern of early driving mutation and the process of tumor evolution vary in individuals (Figure 2E and Figure S6, Supporting Information), presenting the heterogeneity among patients. Briefly, CRLM PDOs successfully capture intra‐ and interpatient heterogeneity.

### 2.4 Gene Expression Profiling of CRLM Organoids

**Figure 3:**
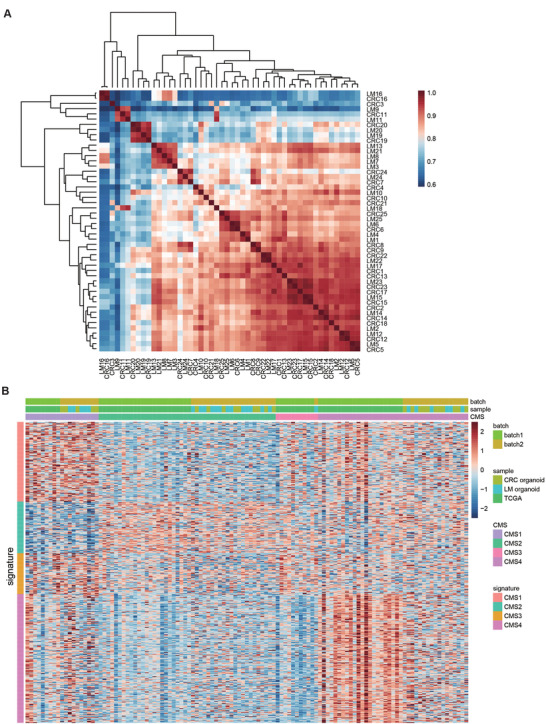
Transcriptomic profiling in CRLM organoids. A) Heat map of Spearman correlation values of CRLM organoids based on RNA‐seq expression data using 15 000 most variable genes and samples were clustered using hierarchical clustering with complete linkage on the correlation matrix. Cells are color‐coded by the Spearman correlation value. B) CRLM organoid RNA‐seq data (50 samples) was normalized and combined with TCGA RNA‐seq data (65 stage IV CRC samples). All CRLM organoids and TCGA samples were redefined by CMS. Within each CMS subtype, all samples are sorted by their mean gene expression for the CMS signature genes associated with that specific subtype. See also Tables S2 and S3 (Supporting Information).

PDOs are three‐dimension cultures, featured by tumor stem cells with different degrees of differentiation, which reflect the characteristics of tumor epithelial cells and eliminate microenvironment (vascular endothelium, immune cells, etc.).^[^ ^18^ ^]^ Therefore, we analyzed the gene expression characteristics of 50 CRLM PDOs by transcriptome sequencing. The correlation heat map of CRLM PDOs was shown in **Figure** **3**A. PDOs from different CRLM patients exhibited much more tumor heterogeneity (for example, P2 and P3 patients). Although PDOs from primary and metastatic lesions in the same CRLM patients also presented different degrees of heterogeneity (for example, P8 and P9 patients), which were transcriptionally more similar to each other than to unrelated organoid lines (for example, P5 and P16 patients).

At present, consensus molecular subtypes (CMSs) provide an integrated framework to capture the intrinsic heterogeneity of CRC.^[^ ^30^ ^]^ The CMS classification has potential clinical value in predicting prognosis and treatment response of CRC patients with at different stages.^[^ ^31^ ^]^ The CMS subtypes of 50 CRLM PDOs and 65 TCGA RNA sequencing (RNA‐seq) tumor tissue samples (TNM stage IV cases) were displayed in Figure 3B (Tables S2 and S3, Supporting Information). CRLM organoid samples were spread across the CMS subtypes, with CMS2 (44%) and CMS4 (34%) being most frequently represented while CMS3 being most rare (Table S3, Supporting Information).

### 2.5 Single Cell RNA Sequencing Profiling in CRLM Organoids

**Figure 4:**
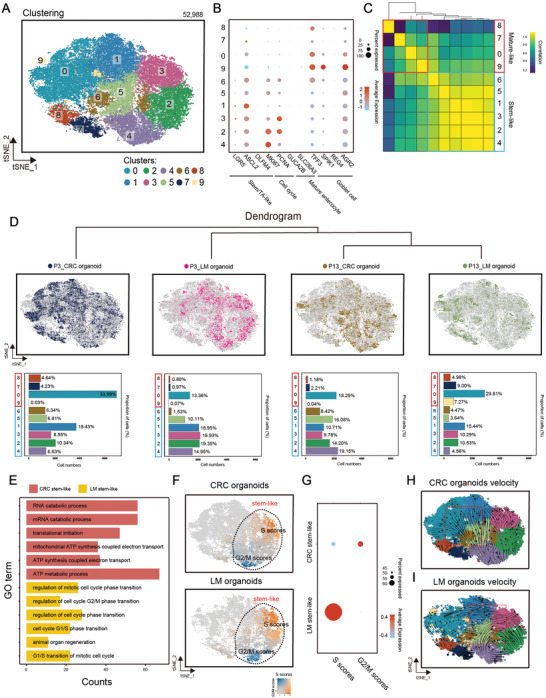
Single cell RNA sequencing profiling in CRLM organoids. A) t‐SNE visualization of 52988 cells from four CRLM organoids (P3_CRC organoid, P3_LM organoid, P13_CRC organoid, and P13_LM organoid). Cells are colored according to clusters. B) Dot plot for the expression of marker genes in each cluster. Color represents the mean expression in each cell cluster, and size indicates the fraction of cells expressing marker genes. C) Correlation between the stem‐like clusters and the mature‐like clusters. D) t‐SNE visualization of 52988 cells from different organoid samples (top). Bar plot showing the proportion of cell clusters (bottom). E) Bar plots showing results of gene ontology enrichment analysis for upregulated genes of stem‐like cells in CRC and LM organoids, respectively. F,G) t‐SNE visualization colored by cell cycle gene scores. Dot plot for the expression of marker genes in each cluster. Color represents the mean expression in each cell cluster, and size indicates the fraction of cells expressing marker genes. H,I) RNA velocities of single cells in CRC and LM organoids. See also Tables S4 and S5 and Figures S7–S9 (Supporting Information).

Analysis of bulk cells could only reflect the average profiles, which was limited in dissecting intratumoral heterogeneity (ITH) across different cellular states of CRLM organoids.^[^ ^32^ ^]^ We next performed single‐cell RNA sequencing (scRNA‐seq) to investigate ITH in CRC and LM organoids from 2 patients (P3 and P13 patients). After quality control including doublet removal, we obtained a total of 52988 cells (**Figure** **4**A). Unsupervised clustering using t‐distributed stochastic neighbor embedding (t‐SNE) revealed ten clusters (Figure 4A). Based on the known annotations of marker genes from the literature,^[^ ^33^, ^34^ ^]^ we found that six clusters (cluster 1, 2, 3, 4, 5, 6) mainly expressed stem/transit‐amplifying (TA)‐like and cell cycle markers (Figure 4B), while four clusters (cluster 0, 7, 8, 9) mainly expressed mature mucosa phenotype markers (Goblet cells). Correlation analysis highlighted the correspondence between the six clusters, and the four clusters. Therefore, we divided ten clusters of four organoids into two cell lineages, including stem‐like (cluster 1, 2, 3, 4, 5, 6) and mature‐like cells (cluster 0, 7, 8, 9) (Figure 4C). The dendrogram showed that organoids from P13 CRLM patient presented strong correlation (Figure 4D and Figure S8, Supporting Information). Quantification analysis of clusters showed that P3 LM organoid mainly contained stem‐like cells (84.82%), compared with CRC organoid (57.71%). However, CRC organoid from P13 CRLM patient possessed more stem‐like cells than its LM organoid. This could potentially indicate the significant discrepancy between CRC and LM organoids regarding their spontaneous differentiation. Subsequent CMS classification showed that mean correlation of single cells with the reference CMS gene expression programs^[^ ^35^ ^]^ of all four organoids mainly reflected the CMS2 canonical subtype (Figure S9A, Supporting Information).

Next, we considered the heterogeneity of the same cell lineage from CRC and LM organoids. By gene ontology (GO) enrichment and subsequent gene score analysis (Figure 4E–G), we observed that stem‐like cells from LM organoids upregulated cell cycle activities and biological process related to regeneration, suggesting that stem/TA‐like cancer cells in liver metastases exhibit higher self‐renewal potential than primary CRC. We next sought to illustrate the lineage relationship between stem‐like and mature‐like cells by performing cell trajectory analysis using scVelo (Figure 4H,I and Figure S9B, Supporting Information). A substantial number of stem‐like cells in CRC organoids showed a differentiation tendency toward mature‐like cells (Figure 4H). Intriguingly, mature‐like cells in LM organoids displayed a tendency back to stem‐like cells (Figure 4I), highlighting the self‐renewal potency of stem‐like tumor cells in metastatic disease. Moreover, these interesting patterns were stable in separate samples (Figure S9B, Supporting Information). We further compared the Ki67 positive rate between CRC and LM organoids cultured for the same days and found that the Ki67 positive rate of CRC organoids was significantly lower than that of LM organoids (*p* < 0.01, Figure S7, Supporting Information). These findings suggested that non‐stem‐like cells in liver metastases display the intrinsic capacity to become stem/TA‐like cancer cells, which is consistent with previous studies.^[^ ^36^, ^37^ ^]^ Overall, these detailed analyses of intra‐tumoral states highlighted the disparity among CRLM organoids.

### 2.6 Responses of CRLM Organoids to 5‐Fluorouracil, Irinotecan, and Oxaliplatin

**Figure 5:**
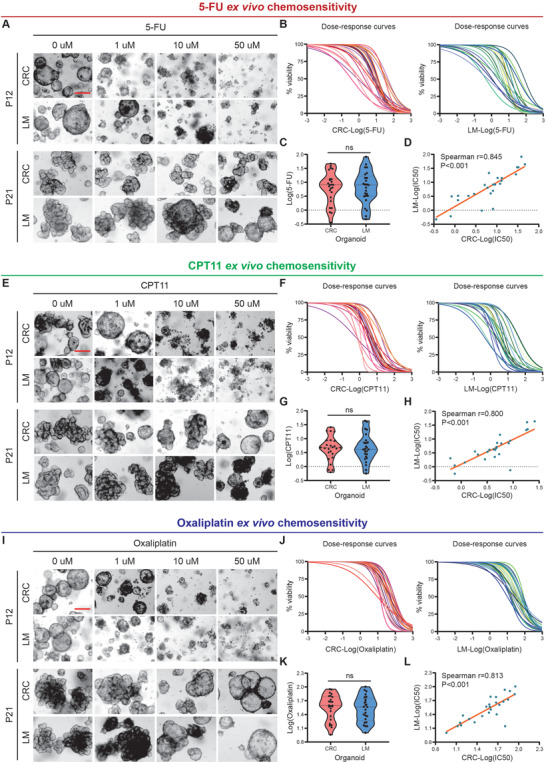
Response of CRLM Organoids to 5‐fluorouracil, irinotecan, and oxaliplatin. A) CRLM organoids dose‐response to 5‐FU (top, representative bright‐field images of organoids sensitive to 5‐FU; bottom, representative bright‐field images of organoids resistant to 5‐FU). B) Ex vivo chemosensitivity of 25 CRC (left) and 25 LM (right) organoids to 5‐FU in the form of dose–response curves are displayed for each CRLM organoid (3 independent experiments for each). C) The standardized IC50 values of CRC and LM organoids were analyzed by paired t‐test to compare 5‐FU sensitivity between them. D) Correlation between the standardized IC50 values of CRC and LM organoids are displayed (two‐tailed Spearman correlation: Spearman *r* = 0.845, *p* ˂ 0.001 for 5‐FU). The linear regression line is plotted. E) CRLM organoids dose‐response to CPT11 (top, representative bright‐field images of organoids sensitive to CPT11; bottom, representative bright‐field images of organoids resistant to CPT11). F) Ex vivo chemosensitivity of 25 CRC (left) and 25 LM (right) organoids to CPT11 in the form of dose–response curves are displayed for each CRLM organoid (three independent experiments for each). G) The standardized IC50 values of CRC and LM organoids were analyzed by paired t‐test to compare CPT11 sensitivity between them. H) Correlation between the standardized IC50 values of CRC and LM organoids are displayed (two‐tailed Spearman correlation: Spearman *r* = 0.800, *p* ˂ 0.001 for CPT11). The linear regression line is plotted. I) CRLM organoids dose‐response to oxaliplatin (top, representative bright‐field images of organoids sensitive to oxaliplatin; bottom, representative bright‐field images of organoids resistant to oxaliplatin). J) Ex vivo chemosensitivity of 25 CRC (left) and 25 LM (right) organoids to oxaliplatin in the form of dose–response curves are displayed for each CRLM organoid (three independent experiments for each). K) The standardized IC50 values of CRC and LM organoids were analyzed by paired t‐test to compare oxaliplatin sensitivity between them. L) Correlation between the standardized IC50 values of CRC and LM organoids is displayed (two‐tailed Spearman correlation: Spearman *r* = 0.813, *p* ˂ 0.001 for oxaliplatin). The linear regression line is plotted. ns, no significance; Red scale bar, 100 µm. See also Table S6 and Figures S10–S12 (Supporting Information).

Regarded as the most important first‐line chemotherapeutic drugs, 5‐FU, CPT11, and oxaliplatin have proven beneficial in the treatment of CRLM. We next separately treated 50 CRLM PDOs in vitro with 5‐FU, CPT11 or oxaliplatin. The chemosensitivity in vitro of PDOs to 5‐FU, CPT11 or oxaliplatin monotherapy varied in CRLM patients (Figure S10, Supporting Information). For example, PDOs from P12 and P21 CRLM patients were sensitive and resistant to 5‐FU (**Figure** **5**A), CPT11 (Figure 5E) and oxaliplatin (Figure 5I), respectively. As shown in Figure 5B,F,J, respectively, the monotherapy sensitivity of 50 CRLM PDOs (25 CRC organoids and 25 LM organoids) to 5‐FU, CPT11 and oxaliplatin were presented by dose–response curve. It showed that the median 50% inhibiting concentration (IC50) of CRLM PDOs was 8.07 × 10^‐6^ m (range from 0.36 × 10^‐6^ m to 82.28 × 10^‐6^ m) for 5‐FU, 4.48 × 10^‐6^ m (range from 0.56 × 10^‐6^ m to 43.46 × 10^‐6^ m) for CPT11 and 38.02 × 10^‐6^ m (range from 9.08 × 10^‐6^ m to 101.30 × 10^‐6^ m) for oxaliplatin (Table S6, Supporting Information). Thus, the CRLM PDOs are heterogeneous in their chemo‐response to 5‐FU, CPT11, and oxaliplatin doses.

Furthermore, we explored the chemosensitivity differences of CRC and paired LM organoids from the same CRLM patient (Figure S11, Supporting Information). There was no significant difference in the drug sensitivity of CRC and paired LM organoids from the same CRLM patient to 5‐FU (Figure 5C), CPT11 (Figure 5G) or oxaliplatin (Figure 5K). IC50 of CRC organoids for 5‐FU (Figure 5D, Spearman *r* = 0.845), CPT11 (Figure 5H, Spearman *r* = 0.800) and oxaliplatin (Figure 5L, Spearman *r* = 0.813) ex vivo chemosensitivity correlated with that of LM organoids from the same CRLM patient (*p* ˂ 0.001 for all treatment conditions).

Given the impact of CMS classification on chemotherapy and prognosis,^[^ ^31^ ^]^ we examined the chemosensitivity of CRLM PDOs to three kinds of drugs in vitro in different CMS subtypes. The previously observed chemotherapy heterogeneity of CRLM PDOs and the chemosensitivity consistency of CRC organoids and paired LM organoids from the same patient were also reflected in different CMS types (Figure S12A–C, Supporting Information). We also observed that CMS1 CRLM PDOs had the worst chemo‐response to 5‐FU, CPT11 and oxaliplatin in vitro (Figure S12D, Supporting Information), although there was no statistical difference, which was consistent with the findings that CMS1 CRC tended to be less sensitive to chemotherapy and had a poor prognosis in metastatic diseases.^[^ ^31^ ^]^

### 2.7 PDOs Predict Chemotherapy Response and Clinical Prognosis of CRLM Patients

As shown in Figure S13 (Supporting Information), 23/25 CRLM patients received FOLFOX or FOLFIRI regimen chemotherapy (P1 and P20 patients did not receive postoperative chemotherapy), including 13 CRLM patients undergoing FOLFOX regimen chemotherapy, 10 patients undergoing FOLFIRI regimen chemotherapy, which also yielded a heterogeneous response to chemotherapy. The median follow‐up time of CRLM patients receiving postoperative therapy was 10.3 months, of which 13 patients developed postoperative disease progression (P2, P3, P4, P6, P7, P9, P10, P11, P17, P18, P19, P21, and P22).

First, according to the combination chemotherapy regimen received clinically by these 23 CRLM patients, we treated 13 CRLM PDOs (P3, P4, P5, P6, P9, P11, P12, P13, P14, P18, P19, P23, and P25) with FOLFOX regimen in vitro, and 10 CRLM PDOs (P2, P7, P8, P10, P15, P16, P17, P21, P22 and P24) with FOLFIRI regimen in vitro. The chemotherapy heterogeneity of CRLM PDOs (Figure S14A,D, Supporting Information) and the chemosensitivity consistency of CRC organoids and paired LM organoids from the same patient were also reflected in the combination chemotherapy regimen (Figure S14B,C, Supporting Information, Spearman *r* = 0.752, *p* = 0.003 for FOLFOX regimen; Figure S14E,F (Supporting Information), Spearman *r* = 0.943, *p* ˂ 0.001 for FOLFIRI regimen, Supporting Information).

**Figure 6:**
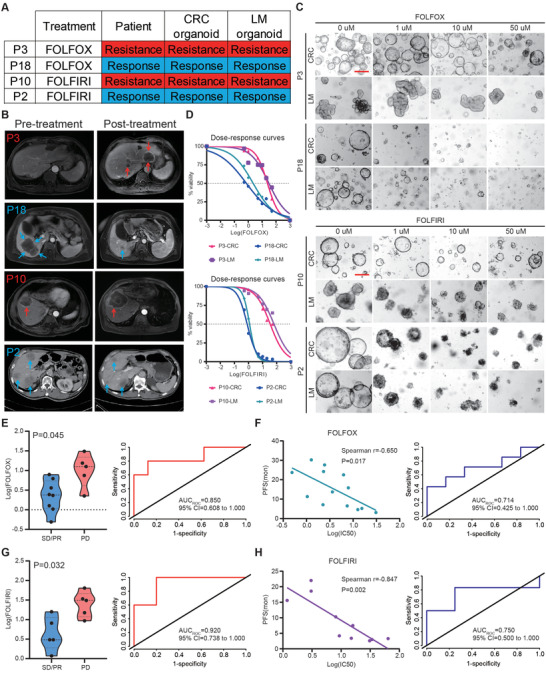
PDOs predict chemotherapy response and clinical prognosis of CRLM patients. A) The table summarizes the results for the selected CRLM organoids and corresponding patients’ drug responses. B) The imaging manifestations of target lesions in 4 CRLM patients before and after treatment, including the progression of lesions in P3 and P10 patients and the regression of lesions in P18 and P2 patients. C) CRLM organoids dose‐response to FOLFOX (top, representative bright‐field images of organoids resistant to FOLFOX (P3 patient); bottom, representative bright‐field images of organoids sensitive to FOLFOX (P18 patient)) and FOLFIRI (top, representative bright‐field images of organoids resistant to FOLFIRI (P10 patient); bottom, representative bright‐field images of organoids sensitive to FOLFIRI (P2 patient)). Red scale bar, 100 µm. D) Ex vivo chemosensitivity of P3 and P18 patient organoids to FOLFOX (top) and P10 and P2 patient organoids to FOLFIRI (bottom) in the form of dose–response curves are displayed (three independent experiments for each). E) The standardized IC50 values of organoids for FOLFOX chemosensitivity from SD/PR patients (*n* = 8) and PD patients (*n* = 5) were compared using a two‐tailed Mann‐Whitney test (left). An ROC curve was plotted to indicate the predictive efficacy of organoids for FOLFOX treatment response (right). F) Correlation between the standardized IC50 values of organoids and progression‐free survival (PFS) for CRLM patients (n = 13) are displayed (Two‐tailed Spearman correlation: Spearman *r* = 0.650, *p* = 0.017 for FOLFOX). The linear regression line is plotted (left). An ROC curve was plotted to indicate the predictive efficacy of organoids for CRLM patients’ clinical prognosis receiving FOLFOX treatment (right). G) The standardized IC50 values of organoids for FOLFIRI chemosensitivity from SD/PR patients (*n* = 5) and PD patients (*n* = 5) were compared using a two‐tailed Mann‐Whitney test (left). An ROC curve was plotted to indicate the predictive efficacy of organoids for FOLFIRI treatment response (right). H) Correlation between the standardized IC50 values of organoids and progression‐free survival (PFS) for CRLM patients (*n* = 10) are displayed (two‐tailed Spearman correlation: Spearman *r* = 0.847, *p* = 0.002 for FOLFIRI). The linear regression line is plotted (left). An ROC curve was plotted to indicate the predictive efficacy of organoids for CRLM patients’ clinical prognosis receiving FOLFIRI treatment (right). See also Table S7 and Figures S13 and S14 (Supporting Information).

As illustrated in **Figure** **6**A,B, P3 and P18 CRLM patients received FOLFOX regimen chemotherapy clinically, of which chemotherapy responses were evaluated resistant and responsive by radiography, respectively; P10 and P2 CRLM patients received FOLFIRI regimen chemotherapy clinically, of which chemotherapy responses were evaluated resistant and responsive by radiography. Our data showed that organoids from P3 and P10 CRLM patients are resistant to FOLFOX and FOLFIRI, respectively, and PDOs from P18 and P2 patients responded to FOLFOX and FOLFIRI, respectively (Figure 6C,D).

Next, we assessed the ability of PDOs to reflect the RECISTs. Of the 13 CRLM patients receiving FOLFOX chemotherapy, 8 were assessed as stable disease (SD)/ partial response (PR) and 5 as progressive disease (PD); Of the 10 CRLM patients undergoing FOLFIRI chemotherapy, 5 were classified as SD/PR and 5 as PD (Table S7, Supporting Information). We quantified chemotherapy responses to FOLFOX or FOLFIRI by calculating the Log(IC50), which were significantly different between PDOs generated from SD/PR versus PD lesions (Mann‐Whitney test, *p* = 0.045 and *p* = 0.032 for FOLFOX and FOLFIRI, respectively; Figure 6E,G). Thus, the CRLM PDOs combinative administration data in vitro highly correlated with patients’ clinical therapeutic response, with 0.850 and 0.920 AUCs for FOLFOX (Figure 6E) and FOLFIRI (Figure 6G) combination therapies.

We then attempted to clarify whether differential FOLFOX or FOLFIRI sensitivity in vitro correlates with clinical prognosis. Progression‐free survival (PFS) was used to evaluate the prognosis of CRLM patients and compared with the chemosensitivity of their corresponding organoids. Log(IC50) for both FOLFOX and FOLFIRI treatments in vitro correlated with PFS of the corresponding CRLM patient (Figure 6F, Spearman *r* = 0.650, *p* = 0.017 for FOLFOX regimen; Figure 6H, Spearman *r* = 0.847, *p* = 0.002 for FOLFIRI regimen). Furthermore, ROCs based on combination therapy produced AUCs of 0.714 (FOLFOX regimen, Figure 6F) and 0.750 (FOLFIRI regimen, Figure 6H), suggesting that PDOs may have predictive value to determine the risk of disease progression for CRLM patients with FOLFOX or FOLFIRI combination chemotherapy.

## 3 Discussion

PDOs and the corresponding tumor carry consistent molecular fingerprints, which account for the possibility of being tumor substitutes, resulting in its continuous development in translational and clinical medicine. Tumor organoids cloning from single tumor cells captured tumor heterogeneity and drug sensitivity at the single‐cell level.^[^ ^38^ ^]^ Recently, researchers have successfully constructed a variety of tumor organoid biobanks, including CRC,^[^ ^14^, ^15^ ^]^ esophageal cancer,^[^ ^39^ ^]^ gastric cancer,^[^ ^20^ ^]^ liver cancer,^[^ ^21^ ^]^ pancreatic cancer,^[^ ^40^ ^]^ lung cancer,^[^ ^41^ ^]^ breast cancer,^[^ ^42^ ^]^ prostate cancer,^[^ ^43^ ^]^ bladder cancer,^[^ ^44^ ^]^ cervical cancer^[^ ^45^ ^]^ and ovarian cancer.^[^ ^46^ ^]^ Although the success rate of tumor organoid construction in vitro largely depends on the tissue sample size, purity, and the access to tumor tissue (biopsy or surgical procedure), the success rate of organoid construction in vitro is usually between 30%–90%, which far exceeds the possibility of establishing stable cancer cell lines from the same tissue.^[^ ^41^ ^]^

In this study, we successfully constructed a living biobank with 50 CRLM organoids, derived from primary tumors and paired liver metastatic lesions, with an overall success rate of 80.6% (86.1% success rate for CRC organoids and 75.0% for LM organoids), which was similar to the previously reported success rate.^[^ ^14^, ^17^ ^]^ Then, we comprehensively analyzed CRLM PDOs from the multi omics levels (histopathology, genome, transcriptome and single‐cell sequencing). It is found that CRLM PDOs retained histopathologic and molecular features of the corresponding tumors from which they were derived, and successfully captured intra‐ and interpatient heterogeneity. Moreover, chemosensitivity test in vitro showed that PDOs are interpatient heterogenetic in their response to monotherapy or combination therapy. Taken together, the tumor and drug heterogeneity of PDOs lay a theoretical foundation for their potential application for personalized medicine.

Relevant studies have shown that organoids from metastatic CRC (mCRC) could predict chemotherapy response.^[^ ^16^, ^17^ ^]^ Vlachogiannis et al.^[^ ^16^ ^]^ found that PDOs could recapitulate patient responses in the clinic and could be implemented in personalized medicine programs. Ooft et al.^[^ ^17^ ^]^ successfully constructed 35 mCRC organoids through biopsy and used them to evaluate the efficacy of chemotherapy. They demonstrated that PDOs could be a predictive tool to prospectively identify mCRC patients who would not benefit from irinotecan‐based palliative chemotherapy. However, their data failed to predict outcome for treatment with 5‐fluorouracil plus oxaliplatin.^[^ ^17^ ^]^ Compared with previous studies, we successfully constructed a living biobank with 50 CRLM organoids derived from primary tumors and paired liver metastatic lesions, which provided a precious biological library for further research on the potential mechanism of liver metastasis from CRC. Indeed, the lack of normal colon and liver organoids weakened the impact of present study to some extent. Such controls could potentially be used to evaluate the tissue‐specific cytotoxicity of chemotherapy in a patient‐specific manner. Although primary and metastatic PDOs from the same CRLM patient are heterogeneous in molecular fingerprints, the performance of drug sensitivity in vitro is highly consistent, which may lay a theoretical foundation for predicting metastatic lesions chemosensitivity by PDOs drug sensitivity from primary lesions, especially in CRLM patients with unresectable lesions. Furthermore, our data show that CRLM PDOs manifest excellent potential to predict the chemosensitivity of FOLFOX or FOLFIRI and the clinical prognosis of patients. Although more prospective, multicenter data are needed to validate our findings, these data indicate that chemosensitivity measured in vitro can be used as a predictive tool to identify the risk of disease progression in CRLM patients.

In conclusion, we have successfully constructed a living CRLM organoid biobank to capture intra‐ and interpatient heterogeneity, which plays a potential role in the prediction for chemotherapy response and clinical prognosis of CRLM patients.

## 4 Experimental Section

### Human Material for Organoid Culture

All tissue collections and experiments were reviewed and approved by the Institutional Review Boards of Fudan University Shanghai Cancer Center (050432‐4‐1212B). Surgical tissues of primary CRC and paired LM were obtained from CRLM patients undergoing enterohepatectomy in the Department of Colorectal Surgery, Fudan University Shanghai Cancer Center. The clinical data of CRLM patients were collected after surgery from the medical records system, including computed tomography (CT) or magnetic resonance imaging (MRI) materials and follow‐up information after surgery. The studies were conducted in accordance with recognized ethical guidelines (Declaration of Helsinki). Informed consent was obtained from all of the participants.

The target tissue samples (normal large intestine tissue, primary CRC tissue, and paired LM tissue) at least 1 cm in diameter were isolated in vitro after simultaneous enterohepatectomy. Following harvesting, each type of tissue was cut into three parts quickly. One part was placed in cold PBS with penicillin/streptomycin (Solarbio, P1400) and transported to the lab in ice box for tumor stem cell isolation and culture. The other two parts were placed into liquid nitrogen and were fixed in 4% paraformaldehyde (Sigma Aldrich, P6148) for sequencing and histopathological analyses, respectively.

### Tumor Cells Isolation and Culture

CRC and paired LM tissues were washed in ice‐cold PBS with penicillin/streptomycin (Solarbio, P1400) for 3 × 5 min, and cut into 1–3 mm^2^ pieces in the sterile dish on ice. Then tissue fragments were washed in ice‐cold PBS with penicillin/streptomycin (Solarbio, P1400) for 3 × 5 min and digested in 10 mL digestion medium containing 10 mL DMEM medium (Hyclone GE Healthcare, SH30243.01), 500 U mL^‐1^ collagenase IV (Sigma‐Aldrich, C9407), 1.5 mg mL^‐1^ collagenase II (Solarbio, C8150), 20 mg mL^‐1^ hyaluronidase (Solarbio, h8030), 0.1 mg mL^‐1^ dispase type II (Sigma‐Aldrich, D4693), 10 × 10^‐6^ m RHOK inhibitor Y‐27632 (Sigma‐Aldrich, Y0503) and 1% fetal bovine serum on an orbital shaker for 30 min at 37 °C. The suspension was collected, filtered through a 100 µm cell filter and then centrifuged at 200 g for 5 min. Isolated cells were embedded in Matrigel in a well of pre‐warmed 24‐well flat bottom cell culture plate (Costar, 3524). After the Matrigel balls were polymerized, 500 µL of human CRLM culture medium was added. Fresh medium was added every 3 days and tumor organoids appeared after 2–3 d.

### CRLM Organoids Culture

Human CRC and paired LM organoids were photographed at the proper times. For passage of tumor organoids, the Matrigel containing organoids were pipetted into 15 mL centrifuge tube using ice‐cold PBS and washed with centrifugation at 200 g. The suspension was removed and the pellets were resuspended in ice‐cold PBS with pipetting 30–60 times using a 1 mL pipette. The cell mixture was washed and embedded in Matrigel at a 1:2 ratio. The culture medium was changed every 3 d. Cryopreservative medium (serum free) (CELLBANKERTM 2, ZENOAQ, 170905) was used to freeze organoids. 10 × 10^‐6^ m Y‐27632 must be added to CRLM culture medium for organoids resuscitating. Human CRLM organoids culture medium (Advanced DMEM/F12 medium, R‐spondin 1, Noggin, EGF, HEPES, Glutamax, Normocin, Gentamicin/amphotericin B, N2, B27, N‐Acetyl‐L‐cysteine, Nicotinamide, Alk 4/5/7 inhibitor, p38 inhibitor, Gastrin and Prostaglandin E2) were used to culture CRLM organoids. Details regarding CRLM organoids culture are provided in the Supporting Information.

### H&E and Immunohistochemistry Staining

Tumor tissues and CRLM organoids were fixed in 4% freshly prepared paraformaldehyde (Sigma Aldrich, P6148‐1KG) followed by dehydration, and paraffin embedding. Sections (4.5 µm in thickness) were cut and hydrated before staining. H&E stains and Immunostaining for Ki‐67, CDX2, *β*‐catenin, CK20, CK‐pan, MLH1, MSH6, MSH2, and PMS2 were performed for all tumor tissues and CRLM organoids. Details regarding primary antibodies used for immunohistochemistry are provided in the Supporting Information.

Briefly, endogenous peroxidase activity was removed by 3% hydrogen peroxide for 10 min at room temperature. Sections were incubated with EDTA antigen retrieval solution for 20 min in steam copper, blocked with 10% donkey serum (Solarbio, SL050) for 1 h, and incubated with primary antibodies (primary antibodies and their concentrations used were listed in the table below) overnight at 4 °C and incubated with secondary antibody (GTVision III Detection System/ Mo & RB, Gene Tech, GK500710) for 1 h at room temperature.

H&E and immunohistochemistry images were acquired on a Zeiss microscope (ZEISS, Imager. M2).

### Whole Exome Sequencing and RNA‐Seq Analysis

CRLM organoids cultured in 24 well plate in good condition were harvested and frozen for DNA extraction and whole exon sequencing. Organoids‐matching tumor DNA was extracted from frozen tissues in liquid nitrogen. Germline DNA was extracted from frozen normal large intestine tissue. CRLM organoids cultured in 24‐well plate in good condition were harvested by Trizol reagent for total RNA extraction following manufacturer's instructions and transcriptomic sequencing analysis. RNA degradation and contamination was monitored on 1% agarose gels. RNA purity was checked using the NanoPhotometer spectrophotometer (IMPLEN, CA, USA). RNA integrity was assessed using the RNA Nano 6000 Assay Kit of the Bioanalyzer 2100 system (Agilent Technologies, CA, USA). Details regarding whole exome sequencing, clonal heterogeneity, tumor evolution analysis, and RNA‐seq analysis are provided in the Supporting Information.

### Single‐Cell RNA Sequencing (scRNA‐Seq) Analysis

Considering the requirements of single‐cell sequencing for cell viability, CRLM organoids with optimum growth state in the selection of samples were mainly considered. CRLM organoids cultured in 24‐well plate in good condition were harvested and digested into single‐cell suspension for sequencing analysis. As per the manufacturer's protocol, single cells were processed through the GemCode Single Cell Platform using the GemCode Gel Bead, Chip and Library Kits (10× Genomics). Cell suspensions of each sample were run in the Chromium Controller with appropriate reagents to generate single cell gel bead‐in‐emulsions for sample and cell barcoding, with a target output of ≈5000 cells for each sample. Amplified cDNA and final libraries were evaluated on an Agilent BioAnalyzer using a High Sensitivity DNA Kit (Agilent Technologies). Libraries were pooled and sequenced on a NovaSeq 6000 (Illumina) at a depth of approximately 400 M reads per sample. Raw sequencing data were converted to FASTQ files with Illumina bcl2fastq, version 2.19.1 and aligned to the human genome reference sequence (GRCH38). The CellRanger (10X Genomics, 4.0.0 version) analysis pipeline was used for sample demultiplexing, barcode processing and single‐cell 3’ gene counting to generate a digital gene‐cell matrix from these data. The gene expression matrix was then processed and analyzed by Seurat package. Seurat‐based filtering of cells based on the number of detected genes per cell (> 500) and the percentage of mitochondrial genes expressed (<15%) was performed. The basic information of scRNA‐seq for organoids is shown in Table S4 (Supporting Information).

For the CMS classification in scRNA‐seq dataset, the Pearson's correlation coefficient value between CMS centroid data and single cells as previously reported was used.^[^ ^34^ ^]^ The CMS centroid data (Table S5, Supporting Information) were obtained from the CMSclassifier package. After calculation, the CMS type with the highest correlation mean was selected.

### Drug Treatments

For drug test, CRLM organoids in good condition were inoculated in 48‐well cell culture plate (Costar, 3548). About 200±50 organoids in 15 µL Matrigel were seeded in each well, covered with 300 µL culture medium. Prepare the solution according to the following drug concentration gradient. 50 × 10^‐6^, 20 × 10^‐6^, 10 × 10^‐6^, 5 × 10^‐6^, 1 × 10^‐6^, 0.5 × 10^‐6^, and 0 × 10^‐6^ m drug concentrations were used for 5‐Fu, CPT11 or oxaliplatin monotherapy. For FOLFOX (5‐Fu:leucovorin:oxaliplatin = 25:5:1) and FOLFIRI (5‐Fu:leucovorin:CPT11 = 25:5:2) treatments, 5‐Fu final concentration was maintained at 50 × 10^‐6^, 20 × 10^‐6^, 10 × 10^‐6^, 5 × 10^‐6^, 1 × 10^‐6^, 0.5 × 10^‐6^, 0 × 10^‐6^ m. Each drug concentration contained three multiple wells. The culture medium containing specific drug concentration was renewed after 3 d.

After 6 d of drug treatment, organoids were photographed (ZEISS, Vert.A1) and organoid cell activity was evaluated by Cell Titer‐Glo‐3D Cell viability assay (Promega, G9683) according to manufacturer's instruction. Graphpad prism 8 (LA Jolla, CA, USA) was used to draw IC50 curve and calculate IC50 value.

### Extraction of Clinical Information for Correlation Analyses to Chemotherapy

Evaluating change of tumor burden before and after treatment is an important feature of clinical evaluation of cancer therapeutics: tumor shrinkage (objective response) and disease progression are useful endpoints in both clinical practice and clinical trials. RECIST guideline (version 1.1)^[^ ^47^ ^]^ was used to evaluate tumor response in this study.

CRLM patient's chemotherapy protocol was made by attending physician according to individual clinical conditions. Chemotherapy response (CR/PR/SD/PD) was determined and recorded by attending physician and radiologist. All CRLM patients in the study had completed the target course of chemotherapy or had disease progression in the course of chemotherapy. The clinical treatment information was retrospectively collected, and carried out the corresponding organoid drug test according to the clinical chemotherapy plan. Progression‐free survival (PFS) was used to evaluate CRLM patient's prognosis after operation. In this study, PFS was measured from the day after the start of systemic treatment until new disease or existing disease progression appeared in the liver lesions. PFS time was determined by the time interval between the start date and the end event.

### Statistical Analysis

Sample size (*n*) for each statistical analysis is provided in the relevant figure legends. For the comparison of drug sensitivity between CRC and LM organoids, the standardized IC50 values of CRC and LM organoids were analyzed by paired t‐test. For the consistency analysis of CRC and LM organoid drug test results, the standardized IC50 values of CRC and LM organoids were analyzed by linear regression. For the correlation analysis of drug test results and CRLM patients’ treatment response, CRLM patients with SD and PR were regarded sensitive to chemotherapy (good response), while CRLM patients with PD were considered resistant to chemotherapy (poor response). The standardized IC50 values of LM organoids were regarded as test variables, and response status to chemotherapy was assigned as state variable. The ROC curve of the above two variables was analyzed, and AUC value was calculated. For the correlation analysis of drug test results and clinical prognosis, the standardized IC50 values of LM organoids and PFS values of CRLM patients were analyzed by linear regression. Similarly, the standardized IC50 values of LM organoids were regarded as test variables, and PFS status was assigned as state variable. The ROC curve of the above two variables was analyzed, and AUC value was calculated. The R software (version 3.6.1, www.r‐project.org) was used for all statistical analyses. All statistics tested 2‐sided, and *p* values <0.05 was regraded statistically significant. *p < 0.05; **p < 0.01; ***p < 0.001; ns, not significant.

## Ethics Approval and Consent to Participate

The studies were performed in accordance with the Declaration of Helsinki and the International Conference on Harmonisation Guideline for Good Clinical Practice. The Ethical Committee and Institutional Review Board of the Fudan University Shanghai Cancer Center reviewed and approved this study protocol. All patients signed written informed consent.

## Conflict of Interest

H.G.Q. and H.C. are scientific founders of D1 Medical Technology. H.C. is an inventor on several patents related to organoid technology. The remaining authors declare no competing interests with relevance to this study.

## Authors Contribution

S.M., P.T., and W.L. contributed equally to this work. J.J.P. and G.Q.H. contributed to conception and design. S.B.M., P.Y.T., and W.Q.L. contributed to the development of methodology. S.B.M., P.Y.T., W.Q.L., L.Z., Y.Q.L., X.H., X.J.M., Y.K.C., Y.C.B., X.F.H., G.X.F., X.Y.X., X.X.R., X.M.L., R.Y.G., S.Z.C., Y.D., T.L., P.Y.M., Q.Z., S.M.W., F.Q.L., Y.W.L., W.Q.S., D.H., J.J.G., Z.Z., and S.J.C. contributed to acquisition of data. S.B.M., P.Y.T., C.H., and W.Q.L. contributed to analysis and interpretation of data. S.B.M. and W.Q.L. contributed to writing of the manuscript. S.B.M., P.Y.T., W.Q.L., C.H., G.Q.H., and J.J.P. contributed to review and revision of the manuscript. J.J.P., G.Q.H., and C.H. contributed to study supervision. All authors approved the final version of the manuscript, including the authorship list.

## Supporting information

Supporting Information

Supplemental Table

## Data Availability

The data that support the findings of this study are available from the corresponding author upon reasonable request.
