# Supplementary material for: Patient‐Derived Organoids from Colorectal Cancer with Paired Liver Metastasis Reveal Tumor Heterogeneity and Predict Response to Chemotherapy
Source: Adv Sci (Weinh). 2022 Sep 4;9(31):2204097. doi: 10.1002/advs.202204097 (PMC9631073; doi:10.1002/advs.202204097)
Supplement: Supplementary file 1 — Supporting Information [file ADVS-9-2204097-s001.pdf]

ADVANCED SCIENCE
Open Access

Supporting Information

for *Adv. Sci.*, DOI 10.1002/advs.202204097

Patient-Derived Organoids from Colorectal Cancer with Paired Liver Metastasis Reveal Tumor Heterogeneity and Predict Response to Chemotherapy

Shaobo Mo, Peiyuan Tang, Wenqin Luo, Long Zhang, Yaqi Li, Xiang Hu, Xiaoji Ma, Yikuan Chen, Yichao Bao, Xingfeng He, Guoxiang Fu, Xiaoya Xu, Xinxin Rao, Xiaomeng Li, Ruoyu Guan, Shengzhi Chen, Yun Deng, Tao Lv, Peiyuan Mu, Qiang Zheng, Simin Wang, Fangqi Liu, Yiwei Li, Weiqi Sheng, Dan Huang, Chen Hu, Jianjun Gao, Zhen Zhang, Sanjun Cai, Hans Clevers, Junjie Peng and Guoqiang Hua**

Supporting Information

Title Patient-derived organoids from colorectal cancer with paired liver metastasis reveal tumor heterogeneity and predict response to chemotherapy

Shaobo Mo, Peiyuan Tang, Wenqin Luo, Long Zhang, Yaqi Li, Xiang Hu, Xiaoji Ma, Yikuan Chen, Yichao Bao, Xingfeng He, Guoxiang Fu, Xiaoya Xu, Xinxin Rao, Xiaomeng Li, Ruoyu Guan, Shengzhi Chen, Yun Deng, Tao Lv, Peiyuan Mu, Qiang Zheng, Simin Wang, Fangqi Liu, Yiwei Li, Weiqi Sheng, Dan Huang, Chen Hu, Jianjun Gao, Zhen Zhang, Sanjun Cai, Hans Clevers, Junjie Peng, Guoqiang Hua**

Corresponding authors:

Guoqiang Hua, Ph.D.

E-mail: guoqianghua@fudan.edu.cn

Junjie Peng, MD, Ph.D.

E-mail: pengjj67@hotmail.com

This Supporting Information file includes:

(1) Supplementary Methods

(2) Supplementary Figures

Fig. S1. Generation of PDOs from CRLM Patients Enrolled in This Study.

Fig. S2. Preservation of CRLM Histopathology in Organoids.

Fig. S3. Conservation of Enterocyte Markers.

Fig. S4. Comparison of Nuclear Mismatch Repair Proteins between CRLM Patient and PDO Samples.

Fig. S5. The Mutational Fingerprint in CRLM Organoids and Corresponding Primary Tumors.

Fig. S6. Riverplots Generated by SuperFreq Analysis Showed The Clonal Evolution of CRLM Organoids Derived from CRC and Paired LM Tumor Tissues.

Fig. S7. Ki67 positive rates of CRC and LM organoids from P3 and P13 CRLM patients cultured for the same days (Day9) were compared. ∗∗p<0.01, Red scale bar, 100 μm

Fig. S8. Correlation analysis based on 2000 variable features via FindVariableFeatures function of Seurat package supported the correspondence between the four samples of organoids.

Fig. S9. Single Cell RNA Sequencing Profiling in CRLM Organoids, related to Figure 4.

Fig. S10. Response of CRLM Organoids to 5-Fluorouracil, Irinotecan, and Oxaliplatin, related to Figure 5.

Fig. S11. Drug Sensitivity in PDOs Derived from 25 CRLM Patients.

Fig. S12. Organoid Drug Sensitivity of Different CMS Subtypes.

Fig. S13. Swimmer's Plot of Each CRLM Patient Whose PDO Has Been Analyzed for Chemosensitivity ex vivo.

Fig. S14. Responses of CRLM Organoids to FOLFOX or FOLFIRI Treatment.

Supplementary Methods

KEY RESOURCES TABLE

REAGENT or RESOURCE	SOURCE	IDENTIFIER
Antibodies		
Rabbit monoclonal anti-Ki-67	Cell Signaling Technology	Cat# 9027; RRID:AB_2636984
Rabbit monoclonal anti-CDX2	Cell Signaling Technology	Cat# 12306; RRID:AB_2797879
Rabbit monoclonal anti-β-Catenin	Cell Signaling Technology	Cat# 8480; RRID:AB_11127855
Rabbit polyclonal anti-Cytokeratin Pan	Proteintech	Cat# 26411-1-AP; RRID:AB_2880505
Rabbit monoclonal anti-Cytokeratin 20	Cell Signaling Technology	Cat# 13063; RRID:AB_2798106
Rabbit polyclonal anti-MLH1	Proteintech	Cat# 11697-1-AP; RRID:AB_2145604
Mouse monoclonal anti-MSH6	Proteintech	Cat# 66172-1-Ig; RRID:AB_2881567
Rabbit monoclonal anti-MSH2	ABclonal	Cat# A8740; RRID:AB_2863598
Rabbit polyclonal anti-PMS2	Affinity Biosciences	Cat# DF4351; RRID:AB_2836719
Biological Samples		
Surgical tissues of CRLM patients	This study	N/A
Chemicals, Peptides, and Recombinant Proteins		
Advanced DMEM/F12	Gibco	Cat# 12634-010
HEPES	Gibco	Cat# 15630080

GlutMAX	Gibco	Cat# 35050-061
Penicillin/streptomycin	Solarbio	Cat# P1400
N2	Gibco	Cat# 17502-048
B27	Gibco	Cat# 17504-044
EGF	Sino Biological	Cat# 50482-MNCH
N-Acetyl-L-cysteine	Sigma-aldrich	Cat# A9165
Nicotinamide	Sigma-aldrich	Cat# N0636
Normocin	invivogen	Cat# ant-nr-2
Gentamicin/AmphoteritinB	Gibco	Cat# R01510
A83-01	Tocris	Cat# 2939
Prostaglandin E2	Sigma-aldrich	Cat# P6532
Gastrin	Sigma-aldrich	Cat# G9145
SB202190	Sigma-aldrich	Cat# S7067
R-spondin-1	Sino Biological	Cat# 11083-HNAS
Noggin	Sino Biological	Cat# 50688-M02H
DMEM medium	Hyclone GE Healthcare	Cat# SH30243.01
Collagenase IV	Sigma-aldrich	Cat# C9407
Collagenase II	Solarbio	Cat# C8150
Bovine serum albumin	BBILife Science	Cat# A600332-0100
Hyaluronidase	Solarbio	Cat# h8030
Dispase type II	Sigma-aldrich	Cat# D4693
Y-27632 dihydrochloride	Sigma-aldrich	Cat# Y0503
Matrigel	Corning	Cat# 356231
TrypLE^{TM} Express	GIBCO	Cat#12605-010
CELLBANKER^{TM} 2	ZENOAQ	Cat#170905
EDTA antigen retrieval solution	BIOTECH WELL	Cat# WH1034
Donkey serum	Solarbio	Cat# SL050
5-Fluorouracil	Selleck	Cat# S1209

Irinotecan	Selleck	Cat# S2217
Oxaliplatin	Selleck	Cat# S1224
Leucovorin	Selleck	Cat# S1236
Phosphate buffered saline	BasalMedia	Cat# B320KJ
Fetal bovine serum	GIBCO	Cat# 10270
Hematoxylin solution	Servicebio	Cat# G1005-1
Eosin solution	Servicebio	Cat# G1005-2
Hydrochloric acid alcohol	Servicebio	Cat# G1039
Critical Commercial Assays		
NEBNext® UltraTM RNA Library Prep Kit	New England Biolabs	Cat# E7530L
GTVisionTM III Detection System/ Mo & RB	Gene Tech	Cat# GK500710
CellTiter-Glo 3D Cell viability assay	Promega	Cat# G9683
Experimental Models: Cell Lines		
Human: CRLM organoids	This study	N/A
Software and Algorithms		
R software	GNU project	https://www.r-project.org/
SPSS 19.0	IBM	https://www.ibm.com/analytics/spss-statisticssoftware
Prism version 8	GraphPad	https://www.graphpad.com/scientific-software/prism/
Other		
TCGA’s Study of Colorectal Carcinoma	NCI	https://www.cancer.gov/about-nci/organization/ccg/research/structural-geno

	mics/tcga/studied-cancers/colorectal

Materials Availability

Distribution of organoids to third parties requires completion of a material transfer agreement and will have to be authorized by the Ethical Committee and Institutional Review Board of the Fudan University Shanghai Cancer Center. Use of organoids is subjected to patient consent; upon consent withdrawal, distributed organoid lines and any derived material will have to be promptly disposed of.

Data Availability

The dataset used during the study are available from the corresponding author on a reasonable request.

CRLM Organoids Culture Media

Regent name	Company	Cat No.	Stock solution	Solvent	Final concentration
Advanced DMEM/F12	GIBCO	12634-010	—	1 ×	1 ×
HEPES	Gibco	15630080	100 ×	—	1 ×
GlutMAX	Gibco	35050-061	100 ×	—	1 ×
Penicillin/streptomycin	Solarbio	P1400	100 ×	—	1 ×
N2	Gibco	17502-048	50 ×	—	1 ×
B27	Gibco	17504-044	100 ×	—	1 ×
EGF	Sino Biological	50482-MNCH	500μg/mL	0.1%BSA/PBS	50ng/mL
N-Acetyl-L-cysteine	Sigma-aldrich	A9165	500mM	ddH2O	1mM
Nicotinamide	Sigma-aldrich	N0636	1M	ddH2O	10mM
Normocin	invivogen	ant-nr-2	500 ×	—	1 ×

Gentamicin/AmphoteritinB	Gibco	R01510	500 ×	—	1 ×
A83-01	Tocris	2939	5mM	DMSO	500nM
Prostaglandin E2	Sigma-aldrich	P6532	100μM	DMSO	10nM
Gastrin	Sigma-aldrich	G9145	100μM	0.1%BSA/PBS	10nM
SB202190	Sigma-aldrich	S7067	30mM	DMSO	3μM
R-spondin-1	Sino Biological	11083-HNAS	50μg/mL	0.1%BSA/PBS	500ng/mL
Noggin	Sino Biological	50688-M02H	10μg/mL	0.1%BSA/PBS	100ng/mL

H&E, Immunohistochemistry, and Immunofluorescence Staining

The primary antibodies used for immunohistochemistry and immunofluorescence were listed below:

Target	Company	Cat No.	Dilution
Ki-67	Cell Signaling Technology	9027	1:400
CDX2	Cell Signaling Technology	12306	1:2000
β-Catenin	Cell Signaling Technology	8480	1:100
CK20	Cell Signaling Technology	13063	1:500
CK-pan	Proteintech	26411-1-AP	1:1500
MLH1	Proteintech	11697-1-AP	1:100
MSH6	Proteintech	66172-1-Ig	1:500
MSH2	ABclonal	A8740	1:100

PMS2	Affinity Biosciences	DF4351	1:100

Whole Exome Sequencing, Mutation and Copy Number Analysis

DNA degradation and contamination (RNA and protein contamination) were monitored on 1% agarose gel, and DNA concentration was accurately measured using Qubit DNA analysis kit in Qubit 2.0 Flurometer (Invitrogen, USA). More than 0.6μg genomic DNA with concentration over 20 ng/μL was used to construct the whole exome sequencing library. Genomic DNA was randomly interrupted by Covaris fragmentation apparatus into fragments with 180-280bp in length. Sequencing library construction and capture experiments were carried out by using Agilent SureSelect Human All Exon kit (Agilent Technologies, CA, USA) following manufacture's recommendations and index codes were added to each sample. After sequencing library was qualified, Illumina Hiseq platform was used for whole exome sequencing and 150bp paired-end reads were generated, according to the effective concentration and data output requirements of the library.

Sequencing data were mapped against human reference genome GRCh37 by Burrows-Wheeler Alignment (BWA)[1] and Samblaster[2] to get the initial comparison results of BAM format. The BAM file is marked and repeated by Samblaster to get the final comparison result of BAM format. If one or a pair of read (s) can have multiple alignment positions on the gene, BWA's processing strategy is to choose the best one from them. If there are two or more best alignment positions, then one from them is randomly selected. We used MuTect software[3] to search for somatic single nucleotide variant (SNV) sites in organoids and paired tissues. Somatic insertion and deletion (InDel) information of organoids and paired tissues was detected by Strelka[4]. Control-FREEC[5] was used to detect somatic copy number variation (CNV) in paired tumor and normal samples. Somatic CNV was based on the depth distribution of reads compared to the reference genome. Considering the base types of 1 bp upstream or downstream of point mutation site, point mutation can be divided into 96 types. Based on the frequency of 96 mutation types in each tumor sample, mutation feature analysis

is to decompose point mutation into multiple different mutation features through Nonnegative Matrix Factorization (NMF) method[6]. Each mutation feature represents one or more tumor mutation process. We compared somatic mutation with the known driver genes in the database to screen out driver genes in organoid and paired tumor tissue samples[7-10]. Significant mutated genes (SMG) take into account the SNV and InDel mutations in somatic cells. MuSiC[11] was used for analysis of SMGs in organoid and corresponding tumor tissue samples.

Clonal Heterogeneity and Tumor Evolution Analysis

SuperFreq[12] was used to track clones across the organoid and tissue. SuperFreq analysis infers and tracks subclones of individual samples by using somatic SNV, InDel and CNV information from cancer exome data. Analysis was performed using variants from GATK UnifiedGenotyper and corresponding BAM files from three groups of normal tissue sample and paired organoid samples from the same patient. Each mutation in a specific sample of each individual is given a clonal value and a specific clone determined by SuperFreq algorithm, thus tracking the clone evolution in the paired organoid samples.

RNA-seq Analysis

A total amount of 1 µg RNA per sample was used as input material for the RNA sample preparations. Sequencing libraries were generated using NEBNext® UltraTM RNA Library Prep Kit for Illumina® (NEB, USA) following manufacturer's recommendations and index codes were added to attribute sequences to each sample. The clustering of the index-coded samples was performed on a cBot Cluster Generation System using TruSeq PE Cluster Kit v3-cBot-HS (Illumia) according to the manufacturer's instructions. After cluster generation, the library preparations were sequenced on an Illumina Novaseq platform and 150 bp paired-end reads were generated. Reference genome and gene model annotation files were downloaded from genome website directly. Index of the reference genome was built using Hisat2 v2.0.5 and paired-end clean reads were aligned to the reference genome. featureCounts v1.5.0-p3 was used to count the reads numbers mapped to each gene. And then FPKM

of each gene was calculated based on the length of the gene and reads count mapped to this gene. Spearman correlation between samples was calculated using the normalized read counts from all 15000 most variable genes and samples were clustered using hierarchical clustering with complete linkage on the correlation matrix. Stage IV CRC cases with gene expression information form TCGA dataset were downloaded from UCSC Xena (https://tcga.xenahubs.net). To subtype all organoid and TCGA samples, we used the consensus molecular subtypes (CMS) of colorectal cancer published by Guinney et al[13].

RECIST Guideline (version 1.1)

Evaluating change of tumor burden before and after treatment is an important feature of clinical evaluation of cancer therapeutics: tumor shrinkage (objective response) and disease progression are useful endpoints in both clinical practice and clinical trials. RECIST guideline (version 1.1)[14] was used to evaluate tumor response in this study. The definition of response criteria in RECIST guideline (version 1.1) was listed below.

Response	Description
Complete Response (CR)	Disappearance of all target lesions. Any pathological lymph nodes (whether target or non-target) must have reduction in short axis to <10 mm
Partial Response (PR)	At least a 30% decrease in the sum of diameters of target lesions, taking as reference the baseline sum diameters
Progressive Disease (PD)	At least a 20% increase in the sum of diameters of target lesions, taking as reference the smallest sum on study (this includes the baseline sum if that is the smallest on study). In addition to the relative increase of 20%, the sum must also demonstrate an absolute increase of at least 5 mm
Stable Disease (SD)	Neither sufficient shrinkage to qualify for PR nor sufficient increase to qualify for PD, taking as reference the smallest sum diameters while on study

Note: the appearance of one or more new lesions is also considered progression

References

[1] H. Li, R. Durbin, *Bioinformatics (Oxford, England)* **2009**, *25*, 1754.
[2] G. G. Faust, I. M. Hall, *Bioinformatics (Oxford, England)* **2014**, *30*, 2503.
[3] K. Cibulskis, M. S. Lawrence, S. L. Carter, A. Sivachenko, D. Jaffe, C. Sougnez, S. Gabriel, M. Meyerson, E. S. Lander, G. Getz, *Nature biotechnology* **2013**, *31*, 213.
[4] C. T. Saunders, W. S. Wong, S. Swamy, J. Becq, L. J. Murray, R. K. Cheetham, *Bioinformatics (Oxford, England)* **2012**, *28*, 1811.
[5] V. Boeva, T. Popova, K. Bleakley, P. Chiche, J. Cappo, G. Schleiermacher, I. Janoueix-Lerosey, O. Delattre, E. Barillot, *Bioinformatics (Oxford, England)* **2012**, *28*, 423.
[6] L. B. Alexandrov, S. Nik-Zainal, D. C. Wedge, P. J. Campbell, M. R. Stratton, *Cell reports* **2013**, *3*, 246.
[7] S. A. Forbes, N. Bindal, S. Bamford, C. Cole, C. Y. Kok, D. Beare, M. Jia, R. Shepherd, K. Leung, A. Menzies, J. W. Teague, P. J. Campbell, M. R. Stratton, P. A. Futreal, *Nucleic acids research* **2011**, *39*, D945.
[8] B. Vogelstein, N. Papadopoulos, V. E. Velculescu, S. Zhou, L. A. Diaz, Jr., K. W. Kinzler, *Science (New York, N.Y.)* **2013**, *339*, 1546.
[9] C. Kandoth, M. D. McLellan, F. Vandin, K. Ye, B. Niu, C. Lu, M. Xie, Q. Zhang, J. F. McMichael, M. A. Wyczalkowski, M. D. M. Leiserson, C. A. Miller, J. S. Welch, M. J. Walter, M. C. Wendl, T. J. Ley, R. K. Wilson, B. J. Raphael, L. Ding, *Nature* **2013**, *502*, 333.
[10] D. Tamborero, A. Gonzalez-Perez, C. Perez-Llamas, J. Deu-Pons, C. Kandoth, J. Reimand, M. S. Lawrence, G. Getz, G. D. Bader, L. Ding, N. Lopez-Bigas, *Scientific reports* **2013**, *3*, 2650.
[11] N. D. Dees, Q. Zhang, C. Kandoth, M. C. Wendl, W. Schierding, D. C. Koboldt, T. B. Mooney, M. B. Callaway, D. Dooling, E. R. Mardis, R. K. Wilson, L. Ding, *Genome research* **2012**, *22*, 1589.
[12] C. Flensburg, T. Sargeant, A. Oshlack, I. J. Majewski, *PLoS computational biology* **2020**, *16*, e1007603.
[13] J. Guinney, R. Dienstmann, X. Wang, A. de Reyniès, A. Schlicker, C. Soneson, L. Marisa, P. Roepman, G. Nyamundanda, P. Angelino, B. M. Bot, J. S. Morris, I. M. Simon, S. Gerster, E. Fessler, E. M. F. De Sousa, E. Missiaglia, H. Ramay, D. Barras, K. Homicsko, D. Maru, G. C. Manyam, B. Broom, V. Boige, B. Perez-Villamil, T. Laderas, R. Salazar, J. W. Gray, D. Hanahan, J. Tabernero, R. Bernards, S. H. Friend, P. Laurent-Puig, J. P. Medema, A. Sadanandam, L. Wessels, M. Delorenzi, S. Kopetz, L. Vermeulen, S. Tejpar, *Nature medicine* **2015**, *21*, 1350.
[14] E. A. Eisenhauer, P. Therasse, J. Bogaerts, L. H. Schwartz, D. Sargent, R. Ford, J. Dancey, S. Arbuck, S. Gwyther, M. Mooney, L. Rubinstein, L. Shankar, L. Dodd, R. Kaplan, D. Lacombe, J. Verweij, *European journal of cancer (Oxford, England : 1990)* **2009**, *45*, 228.

Supplementary Figures

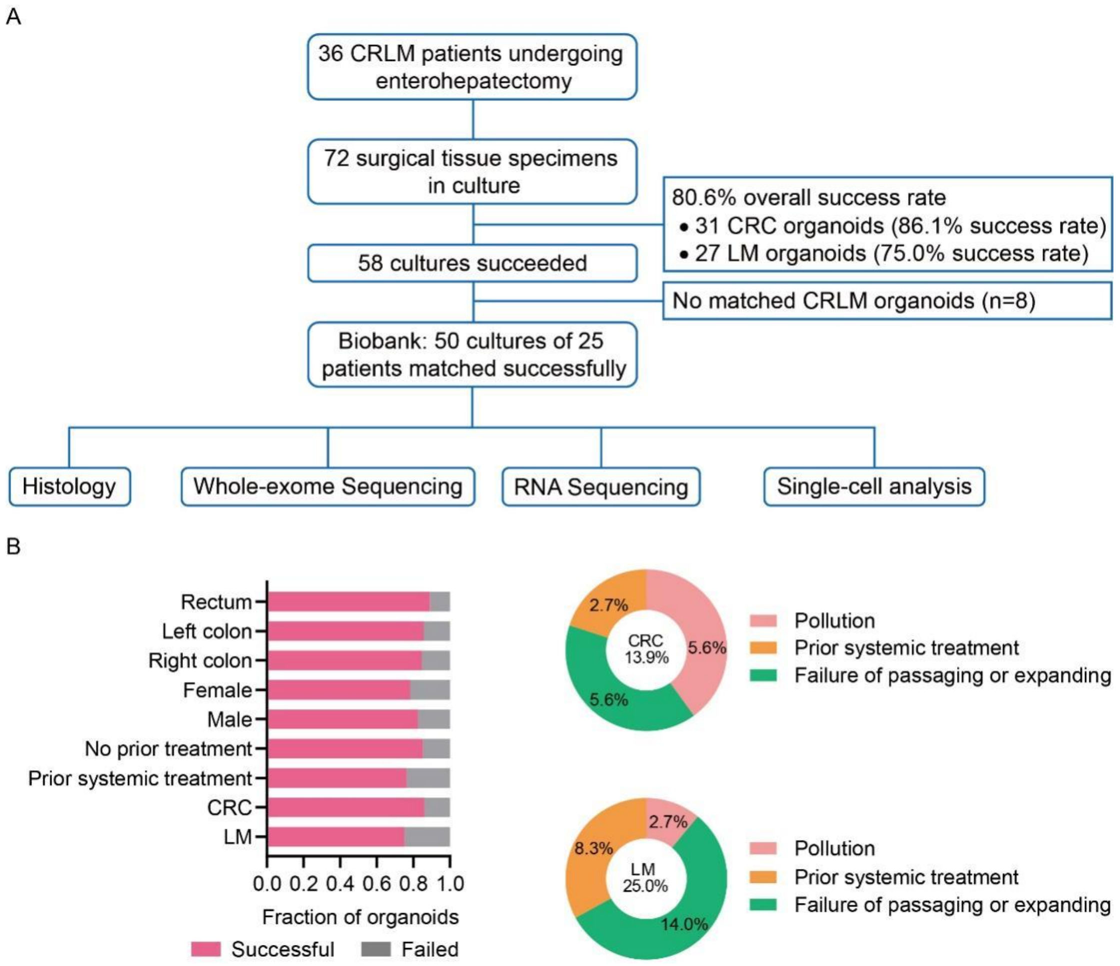

Figure S1. Generation of PDOs from CRLM Patients Enrolled in This Study. A) Flow chart indicating the number of CRLM patients, the number of evaluable patients, the success rate of establishing cultures from patients, and the multiomics analysis of CRLM organoids. B) Overview of clinical parameters in PDO cohorts (left). The pie chart shows the failure factors and proportion of CRC and LM derived organoids (right).

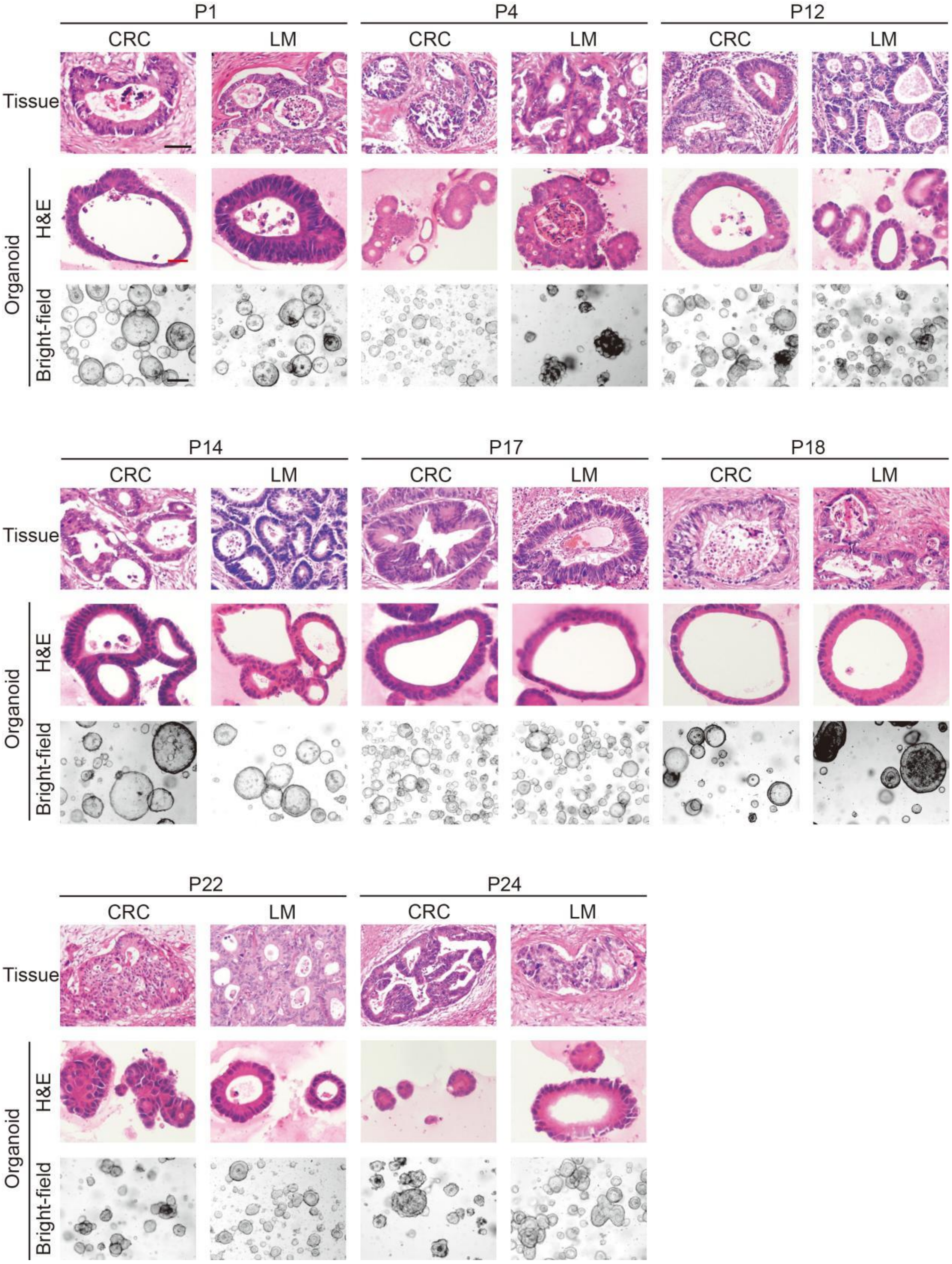

Figure S2. Preservation of CRLM Histopathology in Organoids. H&E comparison of 8 CRC and 8 LM organoids as noted with the corresponding tumor from which they were derived (8 CRLM patients). All representative images of these CRLM organoids in bright field were displayed (bottom). Black scale bar, 200 μm. Red scale bar, 100 μm.

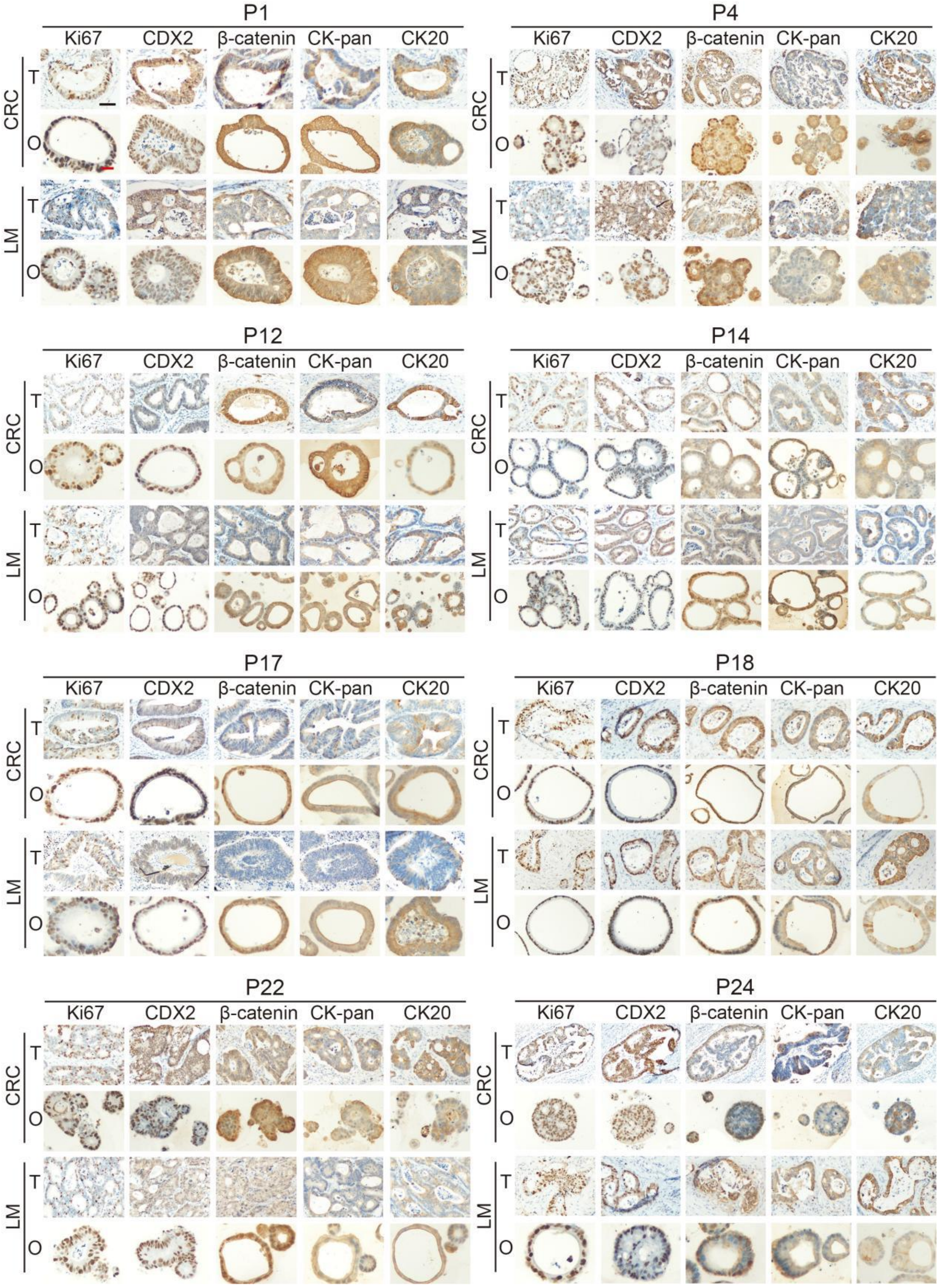

Figure S3. Conservation of Enterocyte Markers. Sixteen PDOs (8 CRC and 8 LM organoids) are compared to their respective tumors for ki-67, CDX2, β-catenin, CK-pan, and CK20 staining. T, tumor tissue; O, organoid. Black scale bar, 200 μm. Red scale bar, 100 μm.

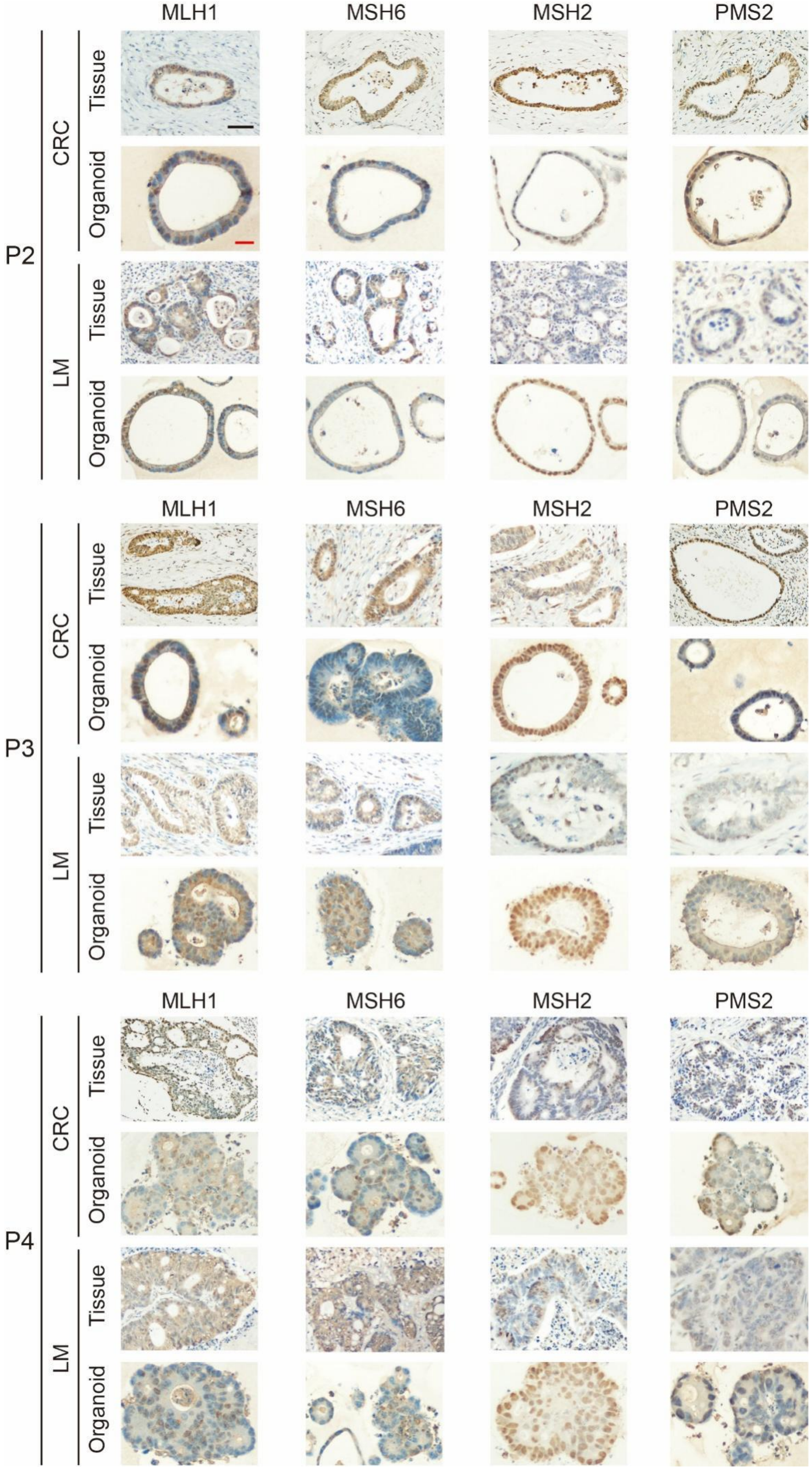

MLH1
MSH6
MSH2
PMS2
P2
P3
P4
CRC
LM
Tissue
Organoid

Figure S4. Comparison of Nuclear Mismatch Repair Proteins between CRLM Patient and PDO Samples. Immunohistochemistry of the nuclear mismatch repair (MMR) proteins MLH1, MSH6, MSH2, and PMS2. Displayed are Six (3 CRC and 3 LM organoids) MMR-proficient PDOs, P2, P3, and P4 CRLM patients. Black scale bar, 200 μm. Red scale bar, 100 μm.

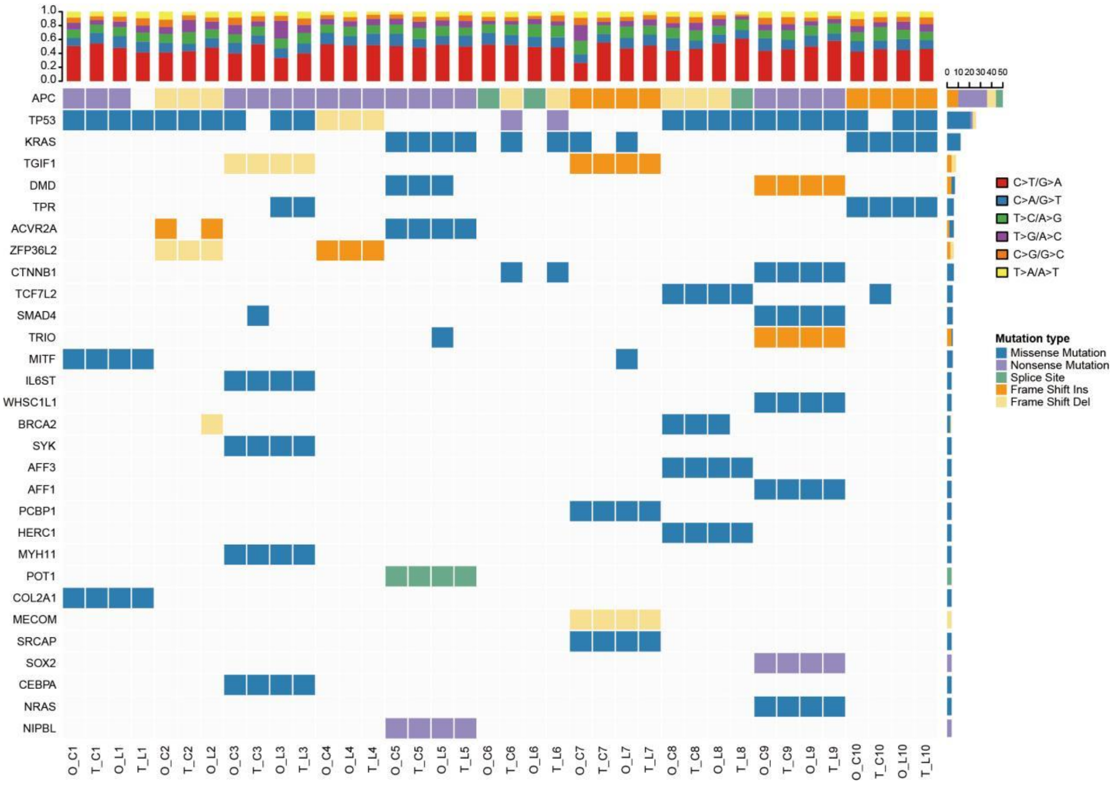

Figure S5. The Mutational Fingerprint in CRLM Organoids and Corresponding Primary Tumors. Overview of somatic tumor driver gene mutations found in CRLM organoids and corresponding primary tumors.

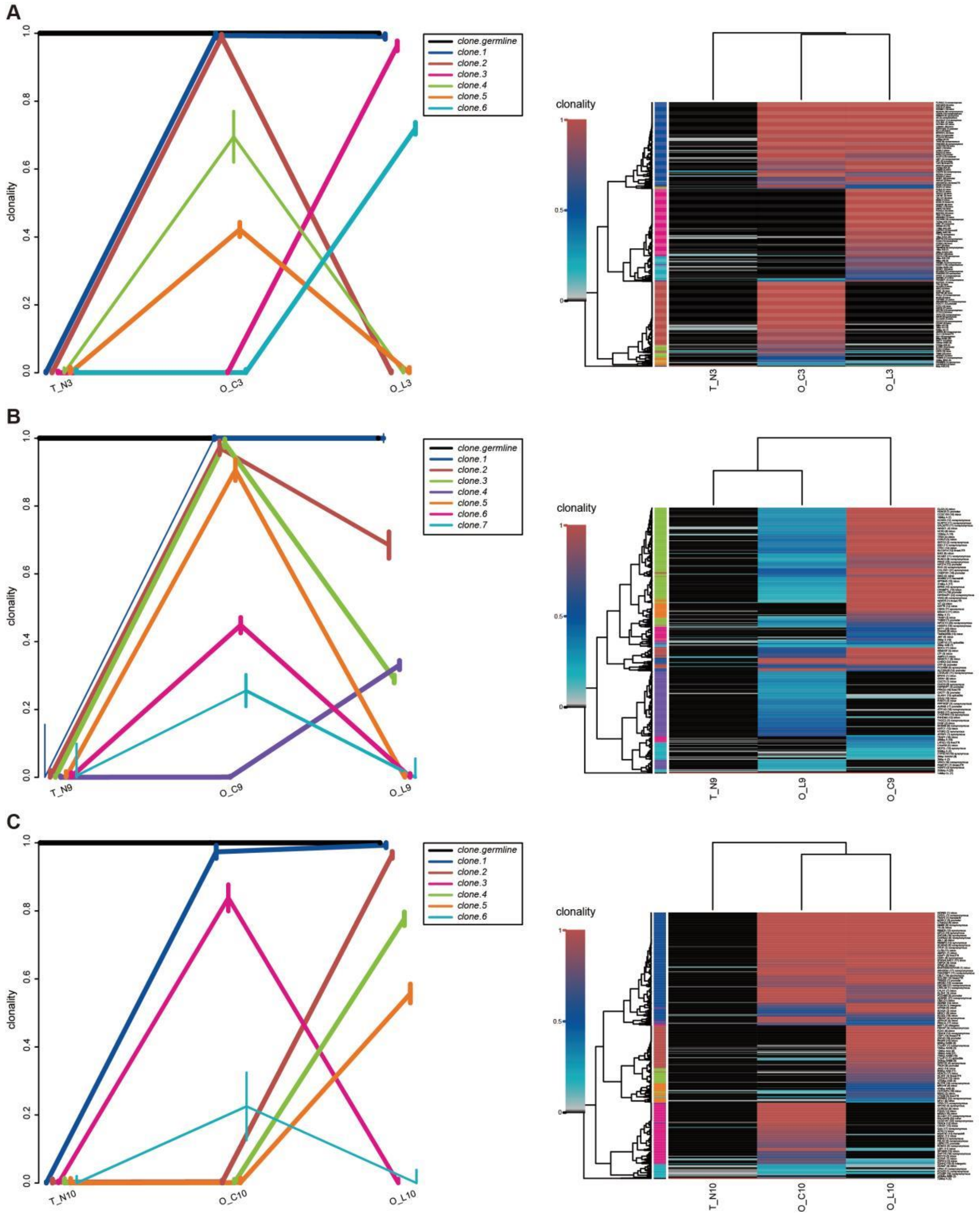

Figure S6. Riverplots Generated by SuperFreq Analysis Showed The Clonal Evolution of CRLM Organoids Derived from CRC and Paired LM Tumor Tissues. The y-axis represents the proportion of tumor cells in each subclone. The different color lines represent different subclones present in CRC and / or LM organoids. The thickness of the color lines corresponds to the number of mutations obtained in the population.

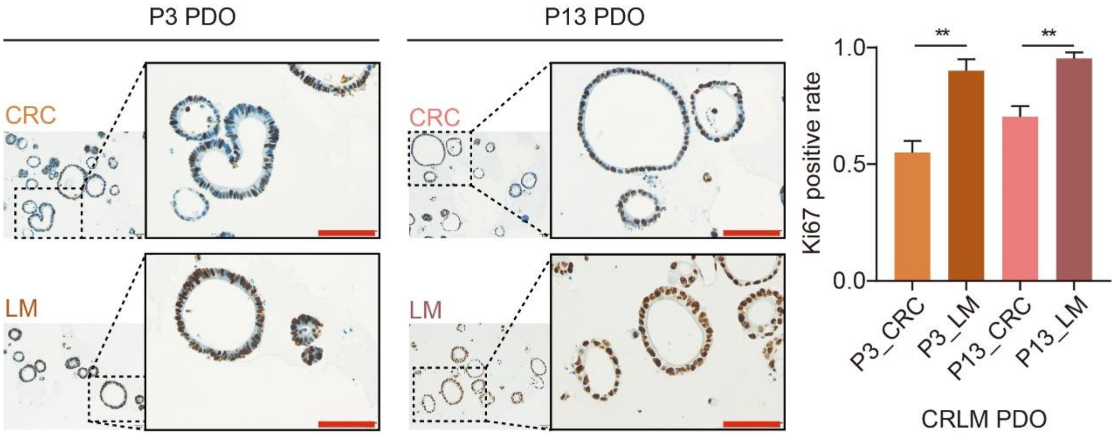

Figure S7. Ki67 positive rates of CRC and LM organoids from P3 and P13 CRLM patients cultured for the same days (Day9) were compared. ∗∗p<0.01, Red scale bar, 100 μm

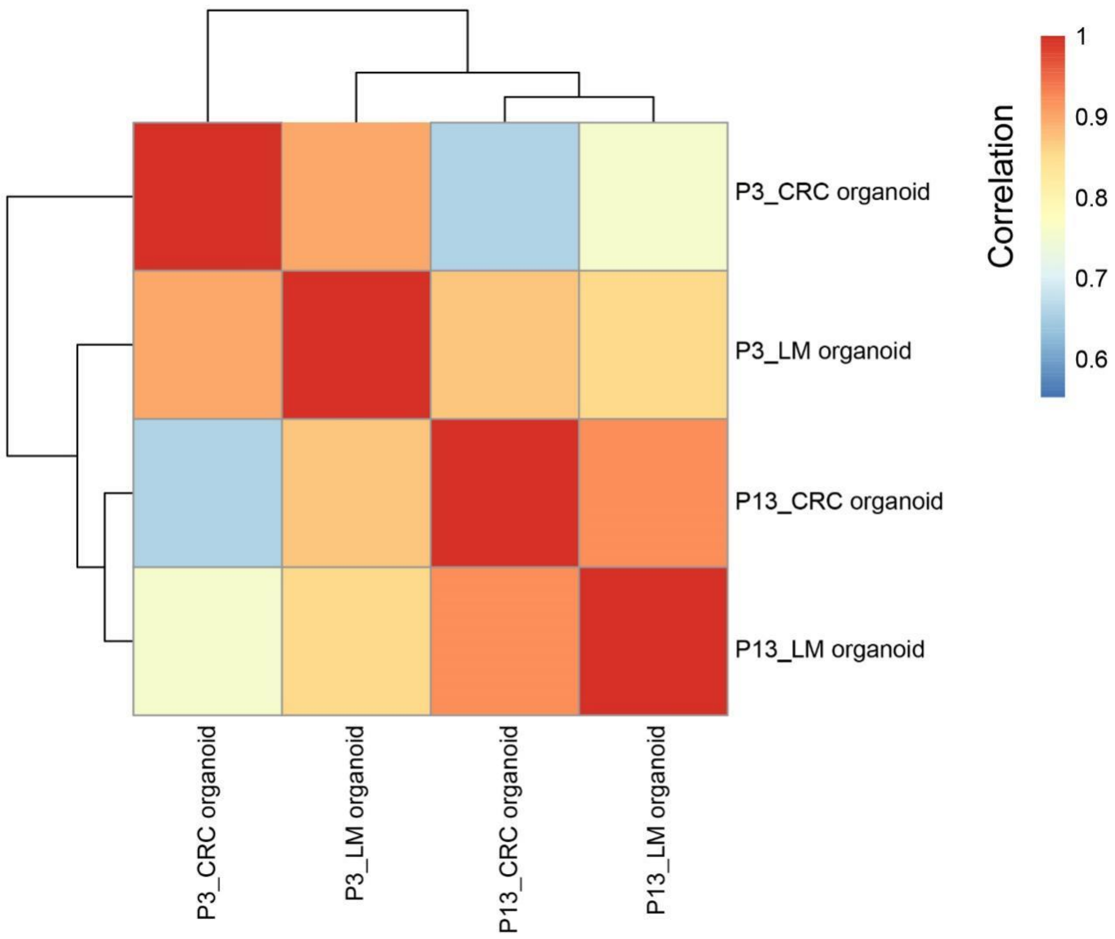

Figure S8. Correlation analysis based on 2000 variable features via FindVariableFeatures function of Seurat package supported the correspondence between the four samples of organoids.

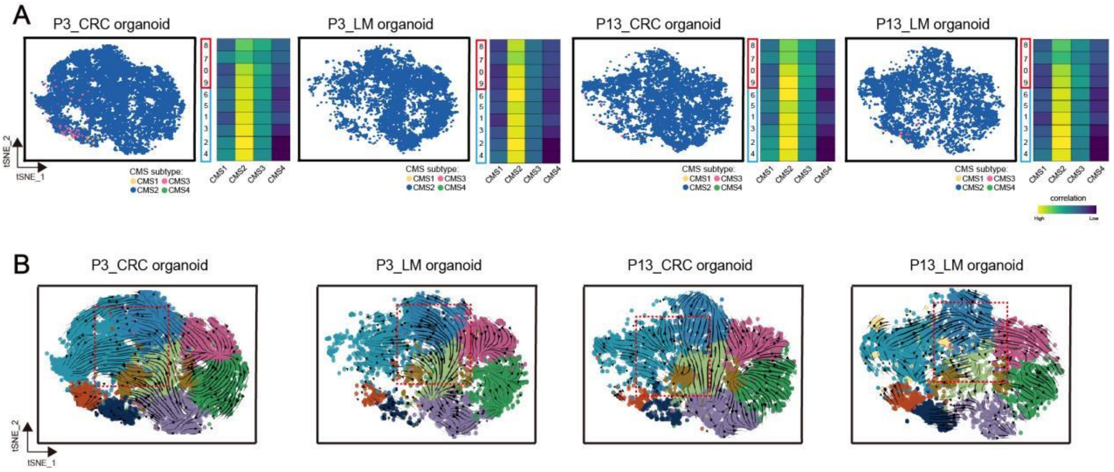

Figure S9. Single Cell RNA Sequencing Profiling in CRLM Organoids, related to Figure 4. A) CMS signatures at the single-cell level (left) and Pearson's correlation to CRC molecular subtype (right) are shown. B) RNA velocities of single cells in each organoid.

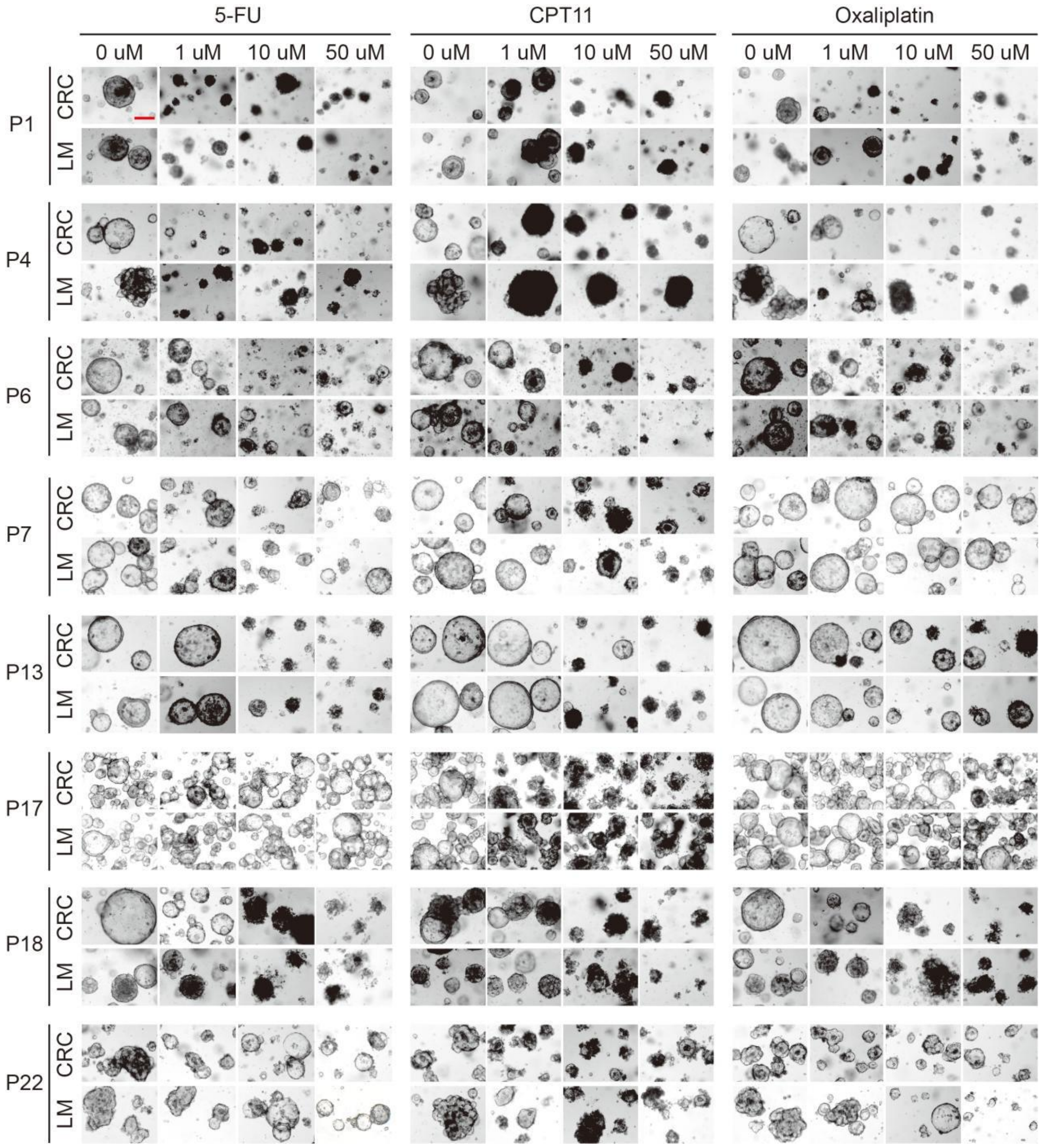

Figure S10. Response of CRLM Organoids to 5-Fluorouracil, Irinotecan, and Oxaliplatin, related to Figure 5. Sixteen CRLM organoids (8 CRC and 8 LM organoids) dose-response to 5-FU, irinotecan, and oxaliplatin. Red scale bar, 100 μm.

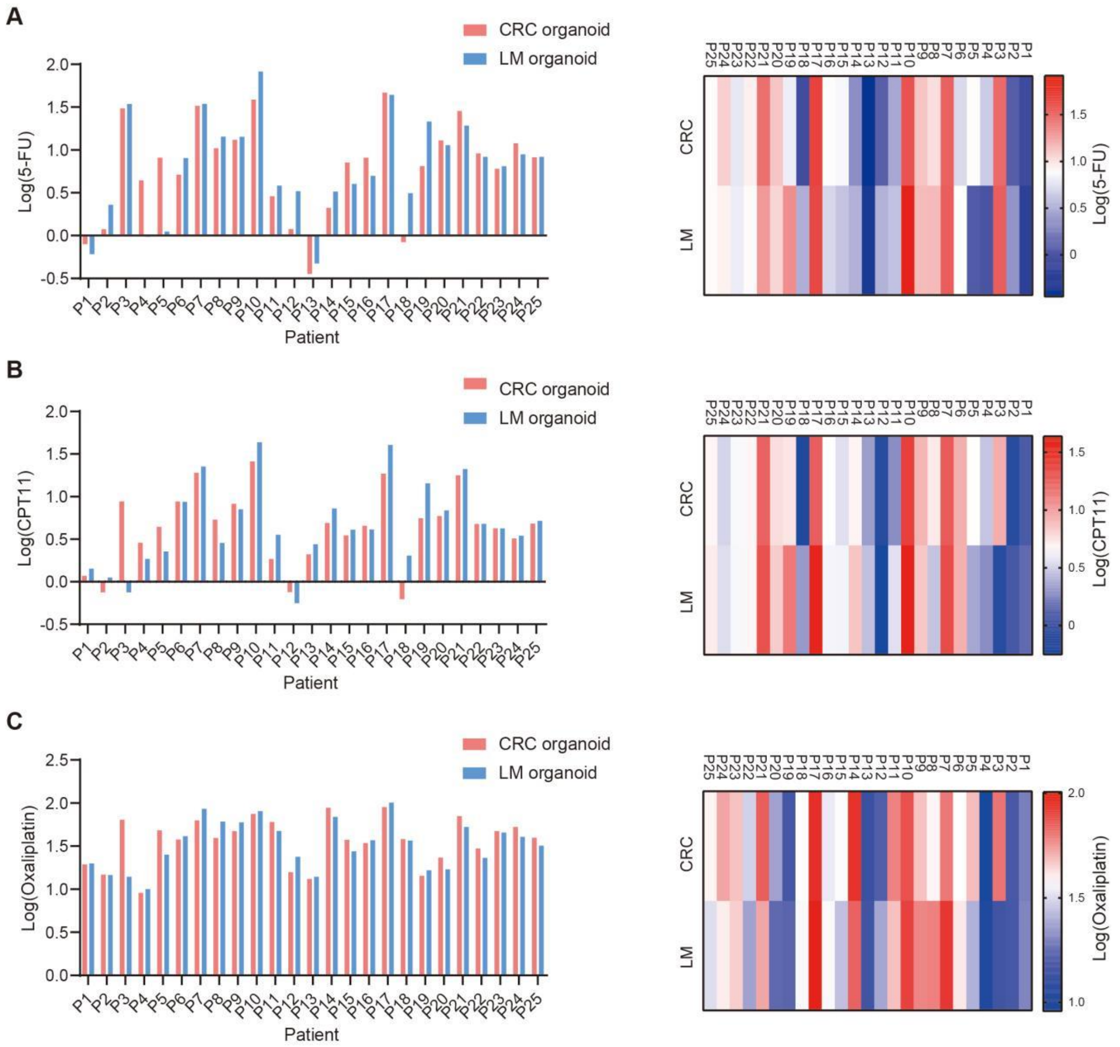

Figure S11. Drug Sensitivity in PDOs Derived from 25 CRLM Patients. The standardized IC50 values of 50 PDOs (25 CRC and 25 LM organoids) from 25 CRLM patients were displayed in the form of bar chart (left) and heat map (right).

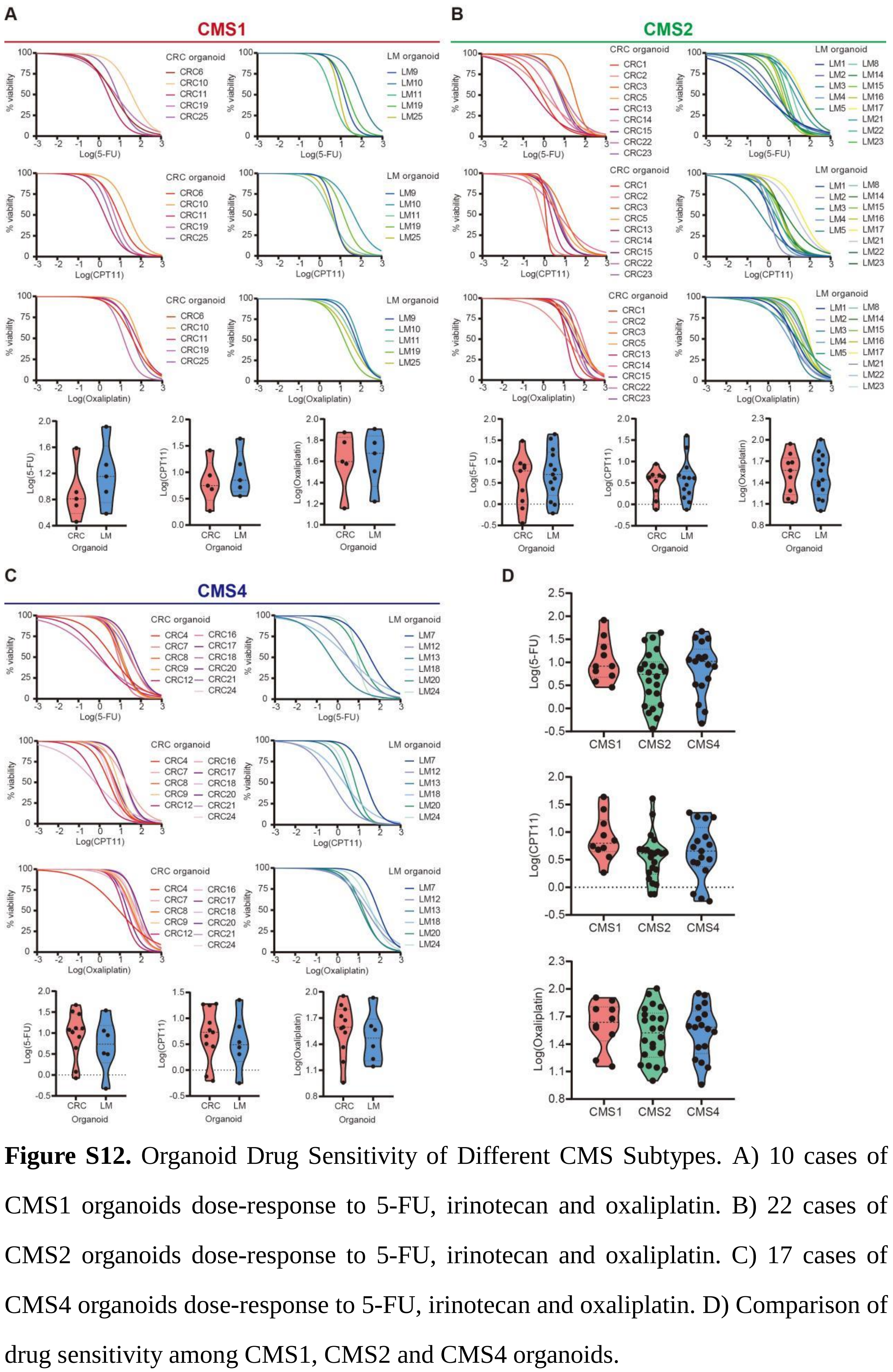

Figure S12. Organoid Drug Sensitivity of Different CMS Subtypes. A) 10 cases of CMS1 organoids dose-response to 5-FU, irinotecan and oxaliplatin. B) 22 cases of CMS2 organoids dose-response to 5-FU, irinotecan and oxaliplatin. C) 17 cases of CMS4 organoids dose-response to 5-FU, irinotecan and oxaliplatin. D) Comparison of drug sensitivity among CMS1, CMS2 and CMS4 organoids.

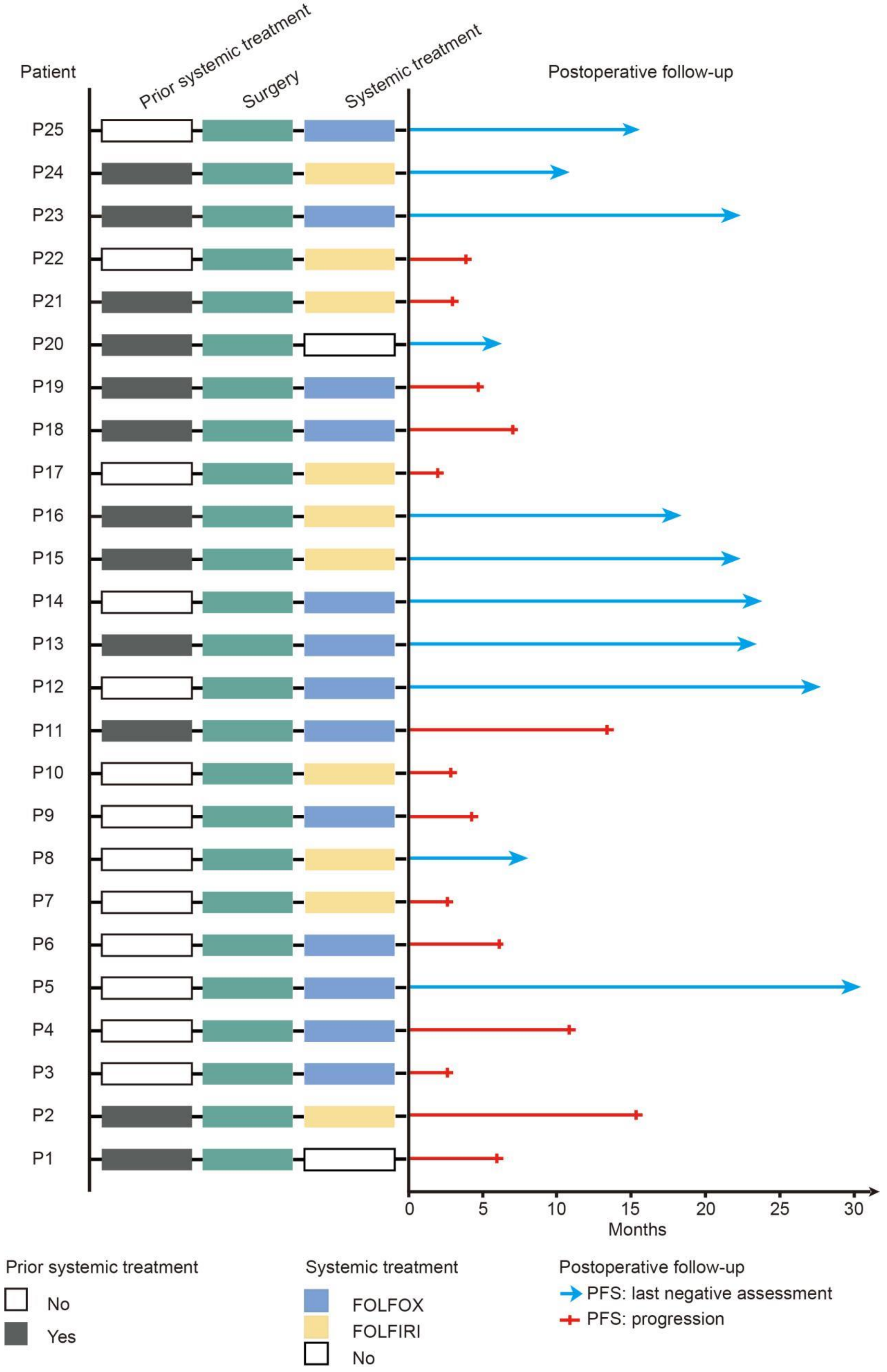

Figure S13. Swimmer's Plot of Each CRLM Patient Whose PDO Has Been Analyzed

for Chemosensitivity *ex vivo*. Clinical treatment information and postoperative prognosis information of 25 CRLM patients have been displayed.

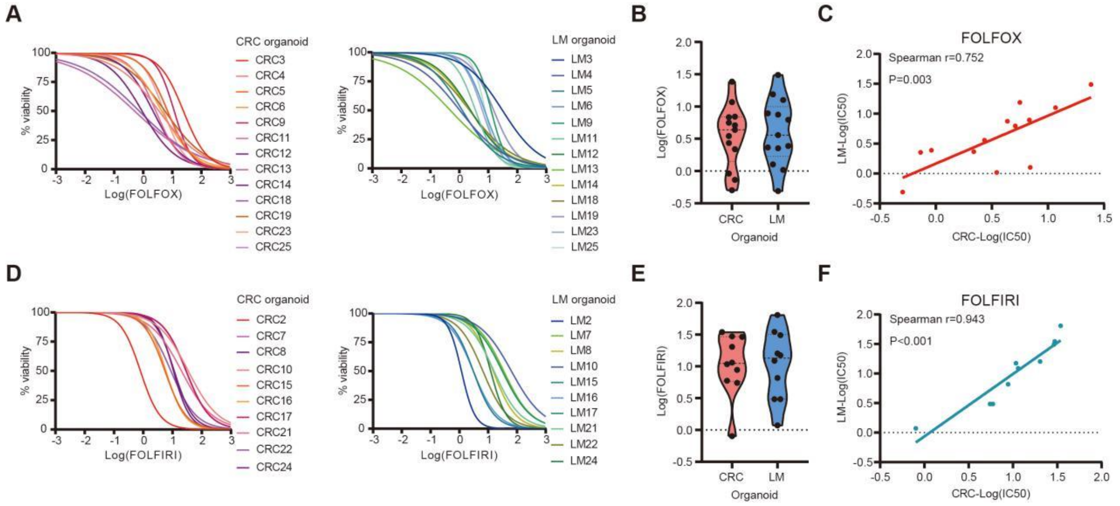

Figure S14. Responses of CRLM Organoids to FOLFOX or FOLFIRI Treatment. A) *Ex vivo* chemosensitivity of 13 CRC (left) and 13 LM (right) organoids to FOLFOX in the form of dose response curves are displayed for each CRLM organoid (3 independent experiments for each). B) The standardized IC50 values of CRC and LM organoids were analyzed by paired t-test to compare FOLFOX sensitivity between them. C) Correlation between the standardized IC50 values of CRC and LM organoids are displayed (Two-tailed Spearman correlation: Spearman r = 0.752, p=0.003 for FOLFOX). The linear regression line is plotted. D) *Ex vivo* chemosensitivity of 10 CRC (left) and 10 LM (right) organoids to FOLFIRI in the form of dose response curves are displayed for each CRLM organoid (3 independent experiments for each). E) The standardized IC50 values of CRC and LM organoids were analyzed by paired t-test to compare FOLFIRI sensitivity between them. F) Correlation between the standardized IC50 values of CRC and LM organoids are displayed (Two-tailed Spearman correlation: Spearman r = 0.943, p<0.001 for FOLFIRI). The linear regression line is plotted.
